# Structure, subunit organization and behavior of the asymmetric Type IIT restriction endonuclease BbvCI

**DOI:** 10.1093/nar/gky1059

**Published:** 2018-11-05

**Authors:** Betty W Shen, Lindsey Doyle, Phil Bradley, Daniel F Heiter, Keith D Lunnen, Geoffrey G Wilson, Barry L Stoddard

**Affiliations:** 1 Division of Basic Sciences, Fred Hutchinson Cancer Research Center, 1100 Fairview Ave. N., Seattle, WA 98109, USA; 2 New England Biolabs, Inc., 240 County Road, Ipswich, MA 01938, USA

## Abstract

BbvCI, a Type IIT restriction endonuclease, recognizes and cleaves the seven base pair sequence 5′-CCTCAGC-3′, generating 3-base, 5′-overhangs. BbvCI is composed of two protein subunits, each containing one catalytic site. Either site can be inactivated by mutation resulting in enzyme variants that nick DNA in a strand-specific manner. Here we demonstrate that the holoenzyme is labile, with the R1 subunit dissociating at low pH. Crystallization of the R2 subunit under such conditions revealed an elongated dimer with the two catalytic sites located on opposite sides. Subsequent crystallization at physiological pH revealed a tetramer comprising two copies of each subunit, with a pair of deep clefts each containing two catalytic sites appropriately positioned and oriented for DNA cleavage. This domain organization was further validated with single-chain protein constructs in which the two enzyme subunits were tethered via peptide linkers of variable length. We were unable to crystallize a DNA-bound complex; however, structural similarity to previously crystallized restriction endonucleases facilitated creation of an energy-minimized model bound to DNA, and identification of candidate residues responsible for target recognition. Mutation of residues predicted to recognize the central C:G base pair resulted in an altered enzyme that recognizes and cleaves CCTNAGC (N = any base).

## INTRODUCTION

Restriction endonucleases (REases) function as an innate form of microbial immune system, distinct from the adaptive form exemplified by CRISPR–Cas nucleases (1). Both act to disrupt invasive DNA molecules such as viral genomes that infect bacterial and archaeal cells. The ∼20-bp target sequences cleaved by CRISPR–Cas nucleases are ‘learned’ from prior exposure and largely recognized via RNA–DNA base pairing (2), whereas the target sequences cleaved by REases are much shorter, are recognized through protein–DNA interactions, and are intrinsic to the structure of each enzyme (3–5). Among proteins that bind to DNA sequence-specifically, REases are exceptional in the high fidelity of target recognition that they achieve. A single base pair substitution in their target sequence usually reduces cleavage activity by many orders of magnitude (6–10), in some cases by too large a reduction to measure (11).

Restriction endonucleases span a wide range of protein folds, catalytic motifs, domain architectures and mechanisms (12,13). This diversity is reflected in a system of classification that divides these enzymes into overlapping groups based upon subunit organization, target characteristics and cleavage positions (14). Type I and III restriction enzymes rely upon ATP-dependent mechanisms that drive independently bound R-M enzyme molecules together, resulting in the formation of synaptic complexes that cleave the DNA sometimes far from the original target binding sites (15–19). Type II enzymes, in contrast, do not translocate and instead cleave within or very close to their target sites. In general, Type II restriction enzymes and their methyltransferase partners are encoded by separate genes, and act independently of one another (20).

Type II REases adopt a variety of structural and functional forms (21). The simplest correspond to monomers or homodimers that recognize palindromic or near-palindromic DNA targets, and cut at equivalent positions within each DNA strand (22–25). More complex Type II enzymes act as homo-tetramers (26–31) or bind to target sites in one structural form, and then oligomerize ‘transiently’ in order to cleave the DNA (32–35). In both cases, binding to two or more target sites simultaneously is often a prerequisite for efficient cleavage (36). Finally, some Type II enzymes contain endonuclease and methyltransferase domains within a single protein chain and, like Type I systems, act in conjunction with a target recognition domain or subunit that determines the sequence specificity of both enzyme activities (37–43). Numerous crystal structures exemplifying enzymes of these kinds have been described. Despite their variety, most use two copies of the same catalytic site to cleave the two strands of the DNA target.

An exception to this latter feature is found among the Type IIT REases, which use different catalytic sites to cleave each strand of the DNA target site. Some Type IIT enzymes comprise two different subunits with one catalytic site each; others comprise a single subunit with two separate catalytic sites (4,44). A structure of a representative Type IIT enzyme has not yet been described, and we report the first one here—that of the best characterized such enzyme, BbvCI.

**Figure 1.**
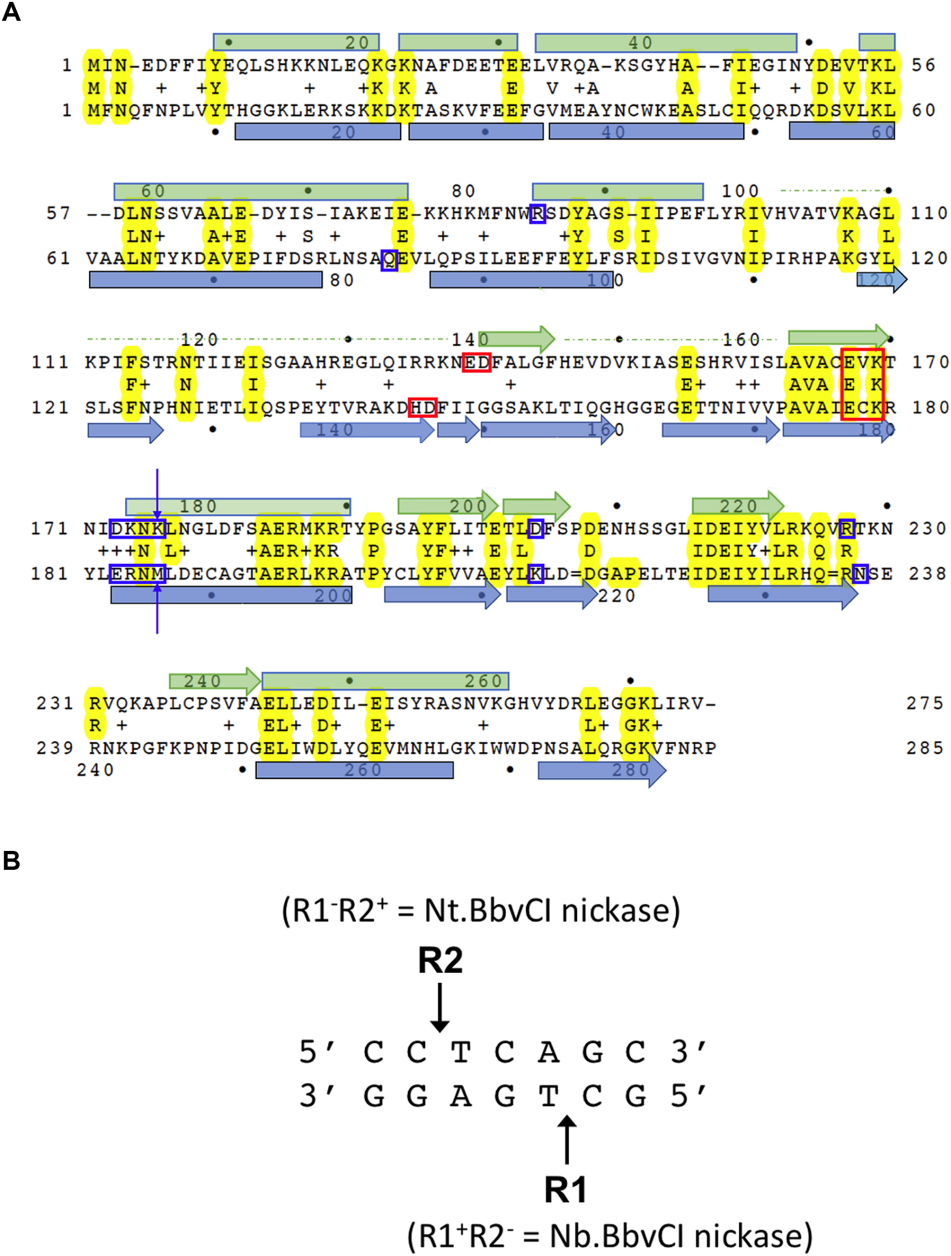
Structure-based sequence alignment of R1 and R2 subunits of BbvCI restriction endonuclease. (**A**) Structure-based amino acid sequence alignment of the BbvCI subunits. Residues that form the PD-(D/E)XK catalytic site motifs are highlighted with red boxes. Residues postulated to be responsible for target-recognition are highlighted with blue boxes. The two residues mutated individually and in combination that alter readout of the central basepair of the target site (Figure 10) are indicated with arrows. (**B**)The seven base pair non-palindromic recognition site of BbvCI, and the positions of strand-cleavage catalyzed by the two subunits.

Identified originally in *Geobacillus brevis*, BbvCI recognizes and cleaves both strands of the asymmetric, 7-bp target sequence 5′-CC|TCAGC-3′ (and its complement, 5′-GC|TGAGG-3′; ‘|’ indicates the positions of cleavage) to generate fragments with 3-base, 5′-overhangs. BbvCI comprises two different subunits, R1 (275 amino acids) and R2 (285 aa), that share approximately 27% sequence identity and are encoded by separate, adjacent genes (45) (Figure 1A; see also Genbank accession no. AY873917). The active enzyme complex contains an equal number of both subunits, neither of which displays DNA-cleavage activity on its own (45). Prior studies indicate that BbvCI exists in a dynamic equilibrium between dimeric, tetrameric, and perhaps larger assemblages (46). The wild-type enzyme cleaves the bottom strand of the recognition site more rapidly than the top strand (45).

Both BbvCI subunits contain a conserved amino acid sequence resembling the…D/EXK… motif that forms the distal part of the catalytic site of many REases (47). Earlier mutagenesis of these residues confirmed their catalytic nature and demonstrated that the site in the R1 subunit cleaves the bottom strand of the target site, while the site in the R2 subunit cleaves the top strand (Figure 1B). These mutations were used to create R1^+^ R2^−^ and R1^−^ R2^+^ ‘nicking’ enzymes that cut only the bottom strand of the target site (designated ‘Nb.BbvCI’), or only the top strand (‘Nt.BbvCI’), respectively (45).

In order to determine the structural organization of BbvCI, and the basis for asymmetric recognition and enzymatic activity, we identified its remaining catalytic site residues, determined the crystal structure of both an R2 homodimer (an unanticipated result caused by loss of the R1 subunit during crystallization at low pH) and, subsequently crystallized the intact holo-enzyme. We examined the solution behavior of the enzyme to account for its unusual behavior during crystallization, and further tested that behavior by constructing tethered, single-chain enzyme variants to prevent dissociation and loss of the R1 subunit. Because we were unable to produce DNA-bound crystals of the enzyme, its mode of DNA-recognition was investigated by homology-based modeling and computational docking, allowing us to implicate potential residues in each subunit that might be involved in DNA sequence-recognition. Mutation of residues predicted to recognize the central C:G base pair resulted in an altered enzyme that recognizes and cleaves CCTNAGC (N = any base).

## MATERIALS AND METHODS

### Gene cloning and site-specific mutagenesis

An *E. coli* recombinant harboring the wild type BbvCI R1 and R2 genes inserted into a derivative of plasmid vector, pUC19, was constructed as described (45). For this study, C-terminal 6xHis-tagged derivatives were made by PCR and inserted into the multiple cloning site of pUC19 between the SphI–SalI or XbaI–SacI sites, and expressed from the resident P_lac_ promoter. Cells harboring these derivatives also harbored the M.DdeI methyltransferase cloned into the compatible plasmid, pACYC184, to protect against endonuclease cleavage. The His-tagged derivatives included individual gene constructs (R1-His alone, or R2-His alone); both genes with only R2 tagged (R1 and R2-His together); both genes with both tagged (R1-His & R2-His, together); and fusions in which the two genes were joined by an arbitrary linker and the resulting composite protein C-terminal tagged (R1-R2-His). All constructs were sequence-verified to confirm their structures.

PCR reactions were performed in 100 μl volumes containing 72.5 μl dH_2_O; 10 microliters 10× ThermoPol buffer (final concentrations: 20 mM Tris–HCl, 10 mM KCl, 10 mM (NH_4_)_2_SO_4_, 2 mM MgSO_4_, 0.1% Triton X-100, pH 8.8 @ 25°C); 5 μl (0.5 μg) template DNA, 5 μl (0.6 μg) of each primer (IDT); and 2.5 μl (5 units) of Deep Vent Exo+ polymerase (NEB). Amplifications were carried out in an Applied Biosystems 2720 thermocycler, typically using the following protocol: 94°C 1 min; 25 cycles of (94°C 30 s; 56°C 30 s; 72°C 1 min); 72°C 7 min; 4°C. Reaction products were purified by 1% agarose-gel (SeaKem, Lonza) electrophoresis, band-excision, gel-dissolution, and spin-column (Zymo Research) cleanup.

Mutations (Tables 1 and 3) were introduced into the BbvCI R1 or R2 genes by PCR using supercoiled plasmid DNA templates and pairs of complementary, fully overlapping, 39-base oligodeoxyribonucleotide primers (IDT) containing the desired single amino acid codon change—typically GCG (alanine) or its complement—in the middle. Following amplification, the linear products were gel-purified, and incubated with 1 μl (20 units) of DpnI for 30 min at 37°C to eliminate carryover of Dam-modified parental templates. Treated products were transformed directly into competent *Escherichia coli* (typically M.DdeI-modified ER2566 or ER3081) and plated onto selective media containing 100 μg/ml ampicillin. After overnight incubation at 37°C, individual transformed colonies were picked, grown in culture and their plasmids purified by plasmid miniprep (Zymo Research). Plasmids were sequenced on an ABI 3730XL DNA Analyzer to confirm that the desired aa change was present, and no others. Single mutants were made using templates carrying the wild type R1 or R2 genes. Double mutants were made using templates carrying previously introduced single mutations, and triple mutants were made using templates carrying previously introduced double mutations.

**Table 1.** Catalytic properties of BbvCI mutants. Enzyme variants were constructed comprising an R1 subunit with one or more point mutations (column 1), paired with a wild type (‘WT’) R2 subunit (column 2) or *vice-versa*. The proteins were partially purified and assayed for DNA-cleavage (column 3) or DNA-nicking (column 4) activity on supercoiled plasmid DNA. ‘—’ indicates no detectable activity; ‘±’ indicates slight activity, and ‘+’, ‘++’, ‘+++’ indicate increasingly higher activities. The specific activities of the double and triple mutants (lower section of table) are given relative to the single mutants: ∼48,000 units per mg protein for R1 (E167G): R2^+^, and ∼42 000 units per mg protein for R1^+:^ R2 (E177G). The specific activities of the tethered R1-R2 single-chain variants (lowest section of table) are given relative to the wild type R1^+^:R2^+^ enzyme: ∼1.3 × 10^6^ units per mg of protein

R1 subunit	R2 subunit	ds cleavage	ss nicking	relative activity (%)	Comment
E95A	WT	—	—		
N138A	WT	++	—		
E139A	WT	+++	—		Non-essential
**D140A**	WT	**—**	**+++**		**Catalytic (bottom strand)**
E147A	WT	+++	—		Non-essential
D149A	WT	+++	—		Non-essential
E155A	WT	+	—		
**E167A**	WT	±	**+++**		**Catalytic (bottom strand)**
**K169A**	WT	**—**	**+++**		**Catalytic (bottom strand)**
E201A	WT	+++	—		Non-essential
E218A	WT	—	—		
					
WT	E93A	+	+		
WT	D144A	+++	—		Non-essential
WT	**D146A**	**—**	**+++**		**Catalytic (top strand)**
WT	E163A	+++	—		Non-essential
WT	E165A	+++	—		Non-essential
WT	**E177A**	**±**	**+++**		**Catalytic (top strand)**
WT	**K179A**	**—**	**+++**		**Catalytic (top strand)**
WT	E211A	**—**	**—**		
WT	D226A	+++	—		Non-essential
WT	E227A	**—**	**—**		
**E167G**	WT	±	**+++**	100% (48 000 u/mg)	R1 single mutant
**E167A+K169A**	WT	—	**++**	∼11%	R1 double mutant
**D140A+E167A+K169A**	WT	—	**+**	∼2%	R1 triple mutant
WT	**E177G**	±	**+++**	100% (42 000 u/mg)	R2 single mutant
WT	**D146A+E177A**	—	**++**	∼3%	R2 double mutant
WT	**D146A+E177A+K179A**	—	**+**	∼0.5%	R2 triple mutant
					
WT	WT	+++	—	100% (1 × 106 u/mg)	Wild-type enzyme
Single-chain fusion WT-X7- WT		±	**—**	<0.1%	7-aa tether
Single-chain fusion WT-X14- WT		++	**—**	∼15%	14-aa tether
Single-chain fusion WT-X21-WT		+++	**—**	∼100%	21-aa tether

The consequences of introduced mutations were examined in two ways. In most cases, mutations were introduced into plasmids carrying both R1 and R2 genes. In these cases, cell-extracts were prepared from the resulting cells and following affinity chromatography, or further purification, the eluates were assayed directly for enzyme activity. Alternately, mutations were introduced into plasmids carrying only the R1 gene or only the R2 gene. In these cases, the resulting subunits were purified individually, and mixed with an equimolar amount of the other, purified, wild-type subunit prior to assaying for activity. If both genes were present in the template and only the R2 gene bore a His-tag (i.e. R1 and R2-His), affinity chromatography led to marked loss of the R1 subunit, we found, and poor recovery of complete enzyme. In order to solve this problem, we switched to templates in which both genes were tagged (i.e. R1-His and R2-His), whereupon recovery of complete enzyme improved.

Plasmid clones carrying fused R1 and R2 genes were constructed using two rounds of PCR amplification. The R2 gene was first amplified using a standard 6xHis reverse primer (R2rev 6xHis) and a forward ‘joining’ primer that, in place of the ATG start codon of R2 gene, coded for an arbitrary 7-aa, 14-aa, or 21-aa polypeptide corresponding to ‘GSSAGSA’, ‘GSSAGSAALINKER’ or ‘GSSAGSAALINKERATETHER’, respectively. In a separate reaction, the R1 gene was amplified using a standard forward primer (R1fwd), and a reverse joining primer that, in place of the stop codon of the R1 gene, included the complement of the arbitrary sequence in the R2 joining primer (see below). The approximately 1-kb PCR products from these reactions were gel-purified, and then combined in equimolar amounts and used as templates for the second round of PCR in which only the standard R1fwd and R2rev 6xHis primers were used. The resulting composite, approximately 2-kb, products were gel-purified, inserted into pUC19, transformed into *E. coli*, and subsequently sequence-verified.

R1-fwd (typical): 5′-GGTTCGGCATGCTAAGGAGGTTTAAAATATGATTAACGAGGACTTTTTTATTTATGA-3′

R2 forward 21-aa joining primer: 5′-GGATCATCCGCAGGATCAGCTGCATAATAAACAAGGAGCGAGCAACCGAGACACACGAGCGATTTAACCAATTTAATCCGTTAGTATAT-3′

R1 reverse 21-aa complementary joining primer:

5′-TCGCTCGTGTGTCTCGGTTGCTCGCTCCTTGTTTATTAGTGCAGCTGATCCTGCGGATGATCCAACACGTATTAACTTCCCTCCCTCCA-3′

R2-rev 6xHis (typical): 5′-GGTTCGGAGCTCTTAATGGTGATGGTGATGGTGTGGTCGATTAAACACTTTA-3′

Note: Underlined sequences correspond to the 3′-portions of the primers that anneal to the 5′-termini of the R1 and R2 genes. The preceding sequences are appendages that introduce cloning sites, translation signals, 6xHis tags, joints, etc.

### Small scale enzyme expression, purification and assays

The BbvCI endonuclease genes were constitutively expressed from the P_lac_ promoter of pUC19. Preparations of the wild-type enzyme for crystallography were purified as described previously (45). Enzyme variants were initially prepared on a small scale as follows. 10–50 ml cultures were grown overnight with shaking at 37°C in Luria broth containing 100 microgram/mL ampicillin. Cells were harvested by centrifugation and re-suspended in 1 ml of lysis buffer (10 mM imidazole, 50 mM Na_2_PO_4_ pH 8.0, 300 mM NaCl) containing 1 mg/ml lysozyme. After 1 h on ice, suspensions were sonicated for 10 s using a model W-375 sonicator (Heat Systems-Ultrasonics) with a micro tip at power 5 and 60% duty cycle, and then clarified by micro-centrifugation at 15 000 rpm for 15′ at 4°C. Clarified extracts were loaded onto Ni-NTA spin columns (Qiagen), processed according to the manufacturer's instructions, and bound protein was eluted in a final volume of 250 μl.

Eluates were assayed for endonuclease activity immediately by titration on supercoiled plasmid DNA substrates. Assay tubes contained 1 μg DNA in 50 μl of NEBuffer 4 (50 mM potassium acetate, 20 mM Tris-acetate, 10 mM magnesium acetate, 1 mM DTT, pH 7.9 @ 25°C) or CutSmart buffer (50 mM potassium acetate, 20 mM Tris-acetate, 10 mM magnesium acetate, 100 μg/ml BSA, pH 7.9 @ 25°C). Up to 5 μl of eluate was added to the first tube in each series, and then serially diluted in 2-fold or square-root 10 (i.e. 3.16)-fold steps through the remaining tubes. Tubes were incubated at 37°C for 1 h, after which 10 μl of 6× Blue gel loading dye was added to each (1× composition: 2.5% Ficoll-400, 11 mM EDTA, 3.3 mM Tris–HCl, 0.017% SDS, 0.015% bromophenol blue, pH 8.0 @ 25°C), and the mixtures analyzed by agarose gel electrophoresis.

### Large scale enzyme expression, purification and crystallization

Larger cultures were grown overnight with shaking on a 1 l scale at 37°C in Luria broth containing 100 μg/ml ampicillin. Cells were harvested by centrifugation and stored frozen at –70°C. Frozen cell pellets were thawed on ice, and re-suspended in 100 ml lysis buffer (10 mM imidazole, 50 mM Na_2_PO_4_ pH 8.0, 300 mM NaCl). Suspensions were disrupted with 3 × 50-s pulses using a model W-375 sonicator with a medium tip at power 7 and 80% duty, and then clarified by centrifugation at 12 000 rpm for 30 min at 4°C.

Nickel affinity columns were prepared in 20 ml Econo-Pacs (Bio-Rad) containing 2.0 ml Ni-NTA Agarose resin (Qiagen) equilibrated with 10–20 ml lysis buffer at 4°C. Clarified cell extracts were applied to the columns followed by 10–20 ml of wash buffer (20 mM imidazole, 50 mM sodium phosphate pH 8.0, 300 mM NaCl) at 4°C. Bound protein was removed with 1.5 ml elution buffer (250 mM imidazole, 50 mM Na_2_PO_4_ pH 8.0, 300 mM NaCl) followed by a further 0.5 ml of the same buffer, all at 4°C. Eluates were either mixed on ice with sterile glycerol to a final concentration of 50% and stored at –20°C, or processed further by Heparin column chromatography, as follows.

5 ml HiTrap Heparin HP columns (GE Healthcare) were equilibrated with 10 column volumes (50 ml) of HPLC buffer (20 mM Tris–HCl pH 8.0 @ 25°C, 0.1 mM EDTA, 1 mM DTT, 5% glycerol) containing 100 mM NaCl. Ni-NTA eluates were diluted to 100 mM NaCl by addition of 2 volumes (4 ml) of HPLC buffer, and loaded onto the HP column. Columns were washed with 2–3 column volumes (10–15 ml) of HPLC buffer, and bound protein eluted with 12 column volumes (60 ml) of HPLC buffer containing an increasing NaCl gradient to 1.0 M. 1 ml fractions were collected and assayed for DNA-cleavage activity. Active fractions (Supplementary Figure S1) were pooled, dialyzed into storage buffer (300 mM NaCl, 20 mM Tris–HCl pH 7.4 @ 25°C, 0.1 mM EDTA, 1 mM DTT, 50% glycerol) and stored at –20°C.

The purified enzyme was dialyzed into 20 mM Tris–HCl, pH 7.5, 50 mM NaCl, 1 mM DTT, concentrated to approximately 16 mg/ml, and stored at –80°C in 50 μl aliquots. Initial crystals (form 1, subsequently found to contain only the R2 subunit in homodimeric complex) were grown in the presence of double stranded DNA (dsDNA) oligos by hanging drop vapor diffusion against a reservoir solution containing 0.8 M ammonium sulfate, 100 mM sodium acetate, pH 4.5, 2 mM CaCl_2_. Subsequently, a second crystal form, found to contain both R1 and R2 subunits in a hetero-tetrameric complex, was also grown in the presence of ds DNA oligos, by equilibration against a crystallization reservoir solution at near-neutral pH (confirmed and adjusted using pH meter and drop-wise addition of NaOH) consisting of 25% PEG 4000 and 100 mM sodium acetate. Despite extensive efforts in the presence of many different ds DNA duplexes, crystals of an enzyme–DNA complex were not obtained.

### Data collection and structure determination

Diffraction data sets (Table 2) were collected on both crystal forms at beamlines 5.0.1 and 5.0.2 of the Advanced Light Source (ALS) synchrotron X-ray facility (Lawrence Berkeley National Laboratory), and at the home X-ray diffraction source at the Fred Hutchinson Cancer Center. The diffraction from crystal forms 1 and 2 extended to 2.2 and 3.8 Å, respectively. The structure of the first crystal form was solved by multiple isomorphous replacement phasing, relying primarily on the heavy-atom derivatization of that crystal via heavy atom soaking in the presence of 200 mM 5-Amino-2,4,6-triiodoisophthalic acid (‘I3C’) for 10 min (Supplementary Figure S2). The initial phases were subsequently augmented with additional phasing information using crystals soaked with tantalum bromide. The lower resolution structure of the enzyme tetramer was solved subsequently, using data from the second crystal form, with phases and initial electron density maps calculated by molecular replacement using, as templates, the R2 model and a partial homology model of R1 based on homology with R2.

**Table 2.** Data and refinement statistics

BbvCI data collection and refinement statistics				
Crystal-ID	R2-I3C	R2-TaBr	R2-Nat	R1-R2 (Tetramer)
Wavelength	1.5418	1.0060	1.0000	1.5418
PDB-ID	6EG7	6M9G	6MAG	6MAF
**Data statistics**				
Space group	*C*222_1_	*C*222_1_	*C*222	*P*32
Unit cell				
*a* (Å)	93.89	86.11	95.71	113.15
*b* (Å)	168.25	173.30	165.71	113.15
*c* (Å)	106.48	107.05	141.18	115.00
α (°)	90	90	90	90
β (°)	90	90	90	90
γ (°)	90	90	90	120
Resolution (Å)	84.12–2.98	47.97–2.35	28.65–2.07	28.4–3.79
Unique reflections	15 456	33 287	62 192	15 367
Redundancy^∗^	13.7(10.9)	21.2(11.8)	4.2(3.7)	5.1(5.0)
Completeness (%)^∗^	93.91(63.60)	99.1(91.4)	96.3(97.7)	98.9(93.8)
*I*/σ*I*^∗^	20.4(6.0)	23.0(2.0)	10.8(1.2)	11.4(1.46)
*R*syn^a^ (%)^∗^	12.6(41.8)	16.0(70.3)	10.7(92.5)	12.9(146.9)
*B*_(iso)_ (Å^2^)	21.89	61.92	44.29	137.6
CC1/2*	0.940	0.877	0.548	0.783
**Refinement statistics**				
Protein atoms	4488 (dimer)	4385 (dimer)	7076 (1.5 dimers)	8547 (tetramer)
Heavy atoms	8 I3C (24 Iodides)	10 Ta_6_Br_12_ 6 Br^−^	—	—
Solvent molecules	9 H_2_O	21 H_2_O	395 H_2_O	—
	1 PEG	2 SO_4_^=^	21 Glycerol	
		1 Acetate	3 PO_4_^=^	
		1 ethyleneglycol	1 Acetate	
*R*-factor^b^ (%)*	21.34(34.2)	19.49(25.2)	19.57(32.0)	28.16(45.7)
*R*-free^b^ (%)*	26.19(63.0)	23.85(30.1)	24.32(34.5)	34.40(47.0)
Rmsd:				
Bond length (Å)	0.0078	0.0132	0.0084	0.0083
Angle (Å)	1.736	2.0135	1.6888	1.7170
Ramachandran (%):				
Core region	92.4	92.62	95.51	88.55
Allowed region	4.17	4.43	3.17	7.92
outliers	3.44^#^	2.95^#^	1.32	3.53^$^

*Highest resolution shell values in parenthesis.

aRmerge = Σ|I_hi_ - <I_h_>|/Σ*I*_h_, where I_hi_ is the ith measurement of reflection h, and <I_h_> is the average measured intensity of reflection h.

^b^R-factor/R-free = Σ_h_|F_h(o_) - F_h(c)_|/Σ_h|_F_h(o)_|. Where R-free was calculated with 5% of the data excluded

from refinement.

^#^Most of the outliers are at the N- or C-terminal disordered region.

^$^Majority of the outliers in the tetramer are in the BbvCI-A subunits.

Data were processed and scaled using the DENZO/SCALEPACK (HKL2000) program package (48). The program Shelx (49) with HKL2MAP graphic interface (50) was used for initial phase determination, and for generation of initial electron density maps. The Refmac5 algorithm (51) and CCP4i graphical interface (52), in the CCP4 program suite (53) were used for refinements. The graphic package COOT (54) was used for model building. Figures were generated with PYMOL (55). Refinement statistics for all four crystal structures are provided in Table 2.

### Computational modeling of the BbvCI/DNA complex

A theoretical model of BbvCI docked to DNA was built using a two-stage optimization procedure implemented in the ROSETTA macromolecular modeling suite (56), with a starting model and distance constraints derived from the unbound structure of the BbvCI heterodimer and the DNA-bound structure of the related enzyme, SgrAI. A zip archive that contains the input files and modified Rosetta source file that was used for the modeling of the BbvCI/DNA complex is provided as supplementary information. For the first optimization stage, a low-resolution representation was used in which protein and DNA residues are represented by backbone heavy atoms and sidechain centroid pseudo-atoms, neglecting sidechain degrees of freedom. The starting model was built by superimposing the SgrAI protein–DNA complex onto the BbvCI heterodimer and copying the DNA into the BbvCI reference frame. The degrees of freedom in the first stage optimization consisted of the rigid-body orientations of the four component chains, two from the BbvCI heterodimer and two from the target DNA duplex. Protein–DNA distance constraints were derived from the SgrAI protein–DNA complex structure in order to reward favorable binding orientation near the catalytic sites, with the DNA register shifted by 1 for constrained residue pairs involving the second chain of BbvCI to reflect the differing cut-site spacing for the two enzymes. The low-resolution stage was followed by an all-atom optimization of the protein–DNA complex in which the protein side chains and DNA backbone as well as the subunit rigid body orientations were flexible.

## RESULTS

### Identification of the BbvCI catalytic sites

The catalytic sites of many REases belong to the ‘PD-(D/E)XK’ nuclease superfamily (47,57). Earlier mutagenesis studies of BbvCI demonstrated that E167-V168-K169 in the R1 subunit, and E177-C178-K179 in the R2 subunit, comprise the distal residues of this motif (Figure 1A). Amino acid substitutions at these positions resulted in either complete enzyme inactivation, or in ‘hemi-active’ enzymes that displayed robust DNA-nicking activity (45). The same studies indicated that the R1 subunit cleaves the bottom strand of the recognition sequence (GC|TGAGG), and the R2 subunit cleaves the top strand (CC|TCAGC) (Figure 1B).

Additional mutants, made for this study, confirmed the catalytic importance of these residues, and demonstrated that D140 (R1 subunit) and D146 (R2 subunit) comprise the proximal residues of the PD-(D/E)XK motifs (Table 1 and Supplementary Figure S3). Thus the catalytic site of the R1 subunit comprises E139-D140…X_26_…E167-V168-K169, and that of the R2 subunit corresponds to H145-D146…X_30_…E177-C178-K179. Double and triple mutants of these catalytic site residues were constructed and assayed. Each mutation reduced specific activity ∼10-fold: the triple mutants were ∼10% as active than the double mutants, and the double mutants were ∼10% as active than the single mutants (Table 1). The positions of residues shown to be critical for catalysis in each enzyme subunit are highlighted in Figure 1A.

Numerous crystal structures of PD-(D/E)XK nucleases reveal that residues within that catalytic motif act in concert with one or more bound magnesium ions to position, orient, and activate a water molecule for in-line nucleophilic attack on the target phosphorus, leading to strand-cleavage by displacement of the 3′-oxygen (58). Our studies indicate that the residues described above are not of equal importance in the BbvCI catalytic sites. Mutation of the aspartate (D140 and D146) or lysine (K169 and K179) residues completely abolishes cleavage by both sites, indicating that these residues are essential for catalysis. Mutation of the glutamate of each site (E167 and E177), in contrast, reduces cleavage greatly, but does not abolish it altogether, indicating that these residues are not absolutely essential (Table 1). Consistent with this observation are reports that the glutamate is absent in some REase catalytic sites, including those from the related NgoMIV-family, and a small neutral or non-coordinating residue such as glycine, alanine, serine or threonine is present, instead (26,59–62). In these cases, an alternative glutamate elsewhere in the chain might act as a substitute (63).

### Crystal structure of a homodimer of R2 subunits

Purified samples of wild-type BbvCI, containing a 1:1 ratio of R1 and R2 subunits yielded crystals under acidic conditions (pH 4.5) in the presence of an excess of duplex DNA oligo containing the target site, and 2 mM calcium chloride. A heavy precipitate also formed in the crystallization solution.

The crystals of the native protein diffracted strongly to 2.1 Å resolution, and belong to space group *C*222, with three subunits (1 +1/2 dimers) in the asymmetric unit. However, after derivatization with heavy atom compounds ‘I3C’ or tantalum bromide, the space group was converted to C222_1_, with only two subunits (one dimer) in the asymmetric unit. We initially calculated MIR phases, built an initial structure, and conducted refinements using datasets corresponding to the latter (heavy-atom) space group, and then subsequently determined the final, higher resolution structure of the native (non-derivatized) protein in the original C222 space group via molecular replacement and phase extension (Table 2).

The resulting electron density maps clearly corresponded to two full-length protein chains, but no density corresponding to DNA was present. Initial attempts to model the density as a heterodimer of R1 and R2 subunits resulted in high values for the refinement *R*-factors, particularly *R*_Free_. Areas of poor fit between modeled protein atoms and electron density, and obvious large residual peaks in difference maps, were evident throughout one of the two subunits during the refinement process.

In contrast, modeling the electron density as a homodimer comprising two copies of the R2 subunit resulted in satisfactory values for *R*_work_ and *R*_free_ (0.189/0.249, respectively), excellent correlation of the model to experimental electron density, and significantly improved geometric quality. Similar attempts to model and refine a homodimer of R1 subunits, instead, further degraded the model's performance and refinement statistics.

These results led us to hypothesize that the R1 subunit(s) of the holo-enzyme had dissociated during crystallization. To test this idea, we compared the behavior of the enzyme at pH 7.5 (the pH of the original storage buffer, which contained a 1:1 stoichiometric balance of both subunits (Supplementary Figure S1) and after acidification to pH 4.5 (the pH of the original crystallization buffer). During size exclusion chromatography at pH 7.5, the enzyme eluted as a single peak at a volume corresponding to a size of at least 120 kD, whereas after acidification and clarification it eluted as a single peak of ∼60–70 kDa (Supplementary Figure S4). In addition, at pH 4.5 the yield of the protein from the sizing column was reduced by approximately 50%, and a single protein band was seen when the peak was analyzed by SDS-PAGE gel electrophoresis.

**Figure 2.**
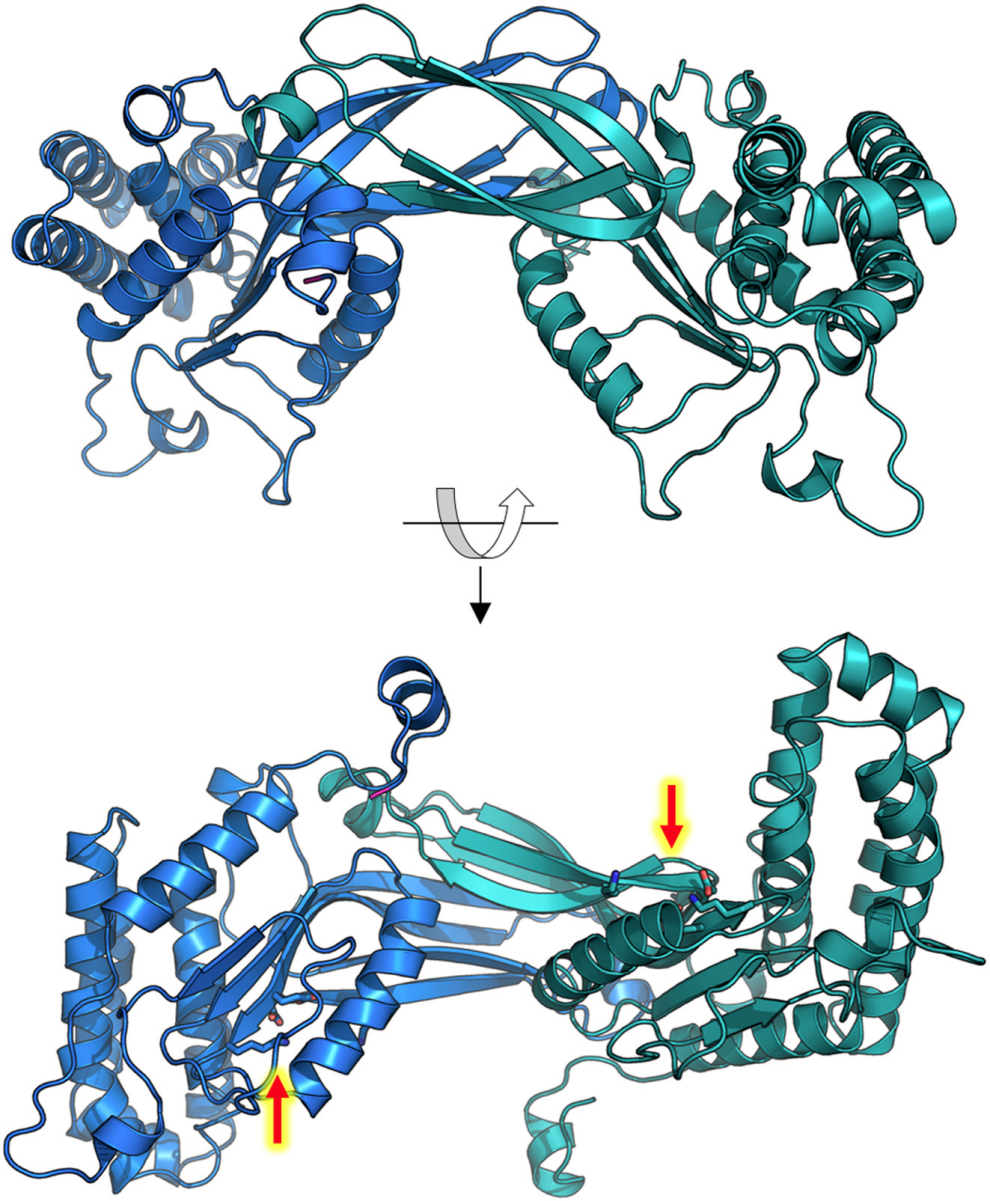
Crystal structure of the homodimer of R2 subunits. The catalytic site residues in each subunit (red arrows, lower panel) are located far apart, on opposite sides of the complex.

The structure of the homodimeric complex of R2 subunits is shown in Figure 2. The first 115, and final 110, residues of each protein chain form a single protein domain of mixed alpha-beta topology built around the alpha-beta-alpha core common to PD-(D/E)XK REases (21). An intervening four-stranded, anti-parallel beta-sheet (residues 116–175) protrudes from this core. This interacts in a domain-swapped manner with its symmetry-mate in the opposing subunit, to form a dimer interface corresponding to an elongated beta-sheet. The dimensions of the homodimer are ∼100 Å × 50 Å × 50 Å. The molecule is saddle-shaped due to deep concave surfaces at the dimer interface, and there is no obvious region of contiguous positive surface charge that might accommodate a DNA duplex of seven or more base pairs.

Within each R2 subunit, the PD-(D/E)XK catalytic site occurs in its customary position, at the termini of adjacent beta-strands in the core domain. However, the two sites in the protein dimer are located ∼45 Å apart from one another–too far away from each other to catalyze double-strand cleavage reactions producing a 3-base overhang.

### Crystal structure of a heterotetrameric assemblage of R1 and R2 subunits

Subsequently, crystals of the BbvCI enzyme were grown at near-neutral pH (*Materials and Methods*). The crystals corresponded to space group P32, with four subunits (i.e. a single enzymatic tetramer) in the asymmetric unit. The crystals diffracted to only 3.8 Å resolution; nevertheless, they produced an obvious molecular replacement solution using the previously determined structure of the R2 homodimer as a search model. In the resulting maps, electron density corresponding to two additional protein chains was clearly present, and this was used to build models of two additional R1 subunits.

While the resolution of this structure is considerably lower than that of the previously determined R2 homodimer, the quality of electron density omit maps was sufficient to visualize the overall quaternary organization of the holoenzyme and significant structural differences between the two subunits in regions where their sequence and structure diverge from one another. A representative region of density is shown in Supplementary Figure S5.

**Figure 3.**
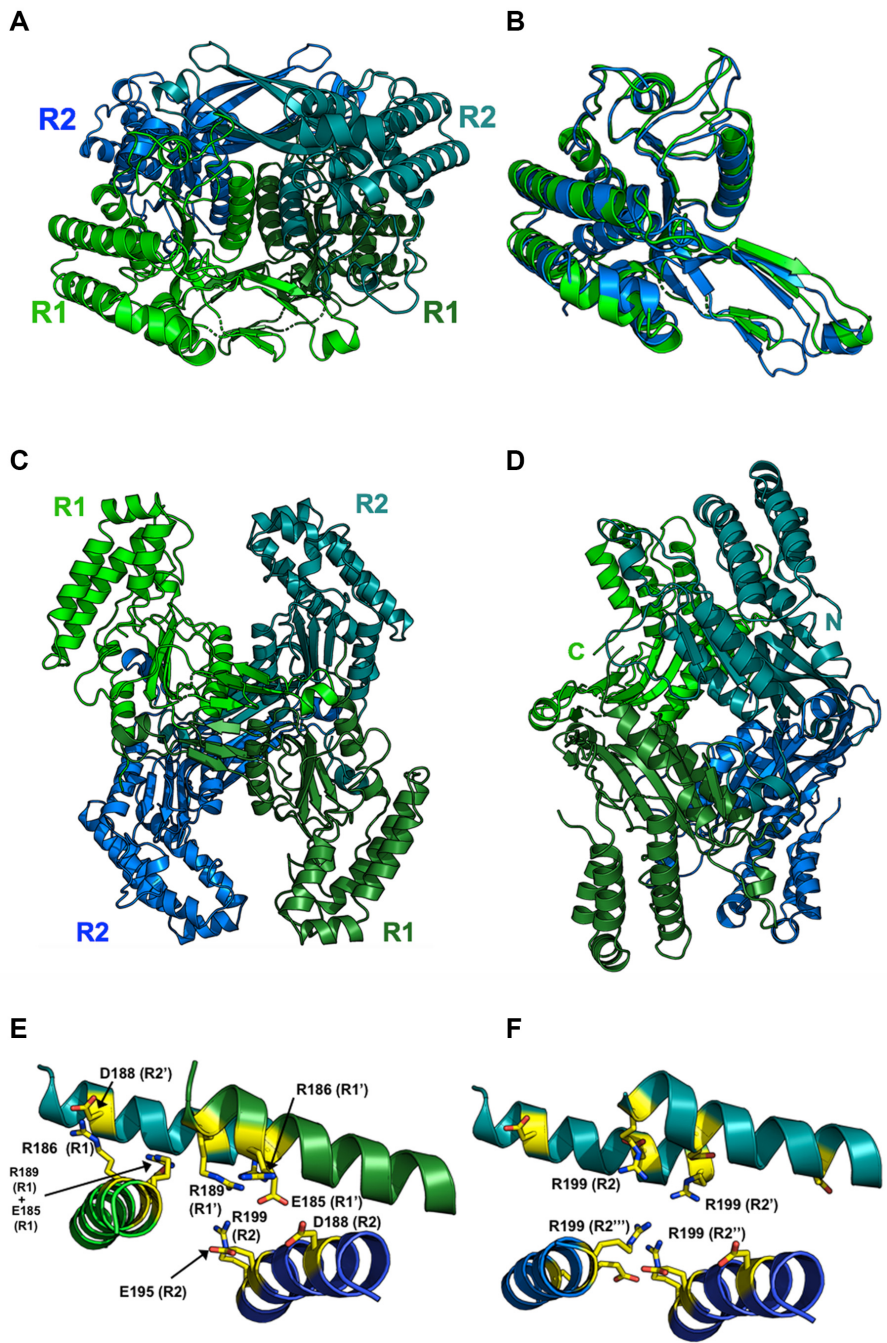
Crystal structure of a heterotetrameric assemblage of 1 R1 and 2 R2 subunits. **Panel A**shows the tetramer with the two R2 subunits in the same color and relative orientation as in Figure 2. **Panel B** shows a superposition of R1 and R2 subunits, with the R1 subunit colored green and in the same orientation as panel A. **Panel C**is related to panel A by a rotation of ∼ 90° about the x-axis. Two deep clefts, formed by neighboring R1 and R2 subunits, are apparent on the top and bottom of the tetramer. Each cleft contains the active site residues of one R1 and one R2 subunit, spaced appropriately to cleave duplex DNA and produce a 5′, 3-base overhang. **Panel D**shows an additional orientation of the heterotetramer rotated by 90° relative to panel C) with the C- and N-termini of the R1 and R2 subunits labeled to indicate the positions bridged by a series of peptide linkers in a separate experiment (enzymatic behavior of those constructs are shown in Figure 7). A second, related pair of termini are located on the opposing side of the construct and were also tethered in that subsequent construct. **Panel E:** The center of the heterotetrameric interface, which is comprised of four equivalent helices (two from the R1 subunits and two from the R2 subunits. Those helices collectively contribute 12 charged residues (3 from each helix) into a highly charged cluster salt-bridges comprised of six arginine residues and six acidic (glu/asp) residues as shown (the net charge is zero). **Panel F:**If we model a homotetramer of R2 subunits only (using the positions of the R1 subunits in the crystal structure shown above as a guide), that cluster is disrupted, gaining a net positive charge and positioning four symmetry-related copies of Arg 199 directly into close contact with one another at the center of the tetramer.

In the resulting hetero-tetramer (Figure 3), the two R2 subunits occupy the same positions and orientations as they do in the R2 homodimer (Figure 3A). The R1 subunits are similar in structure to the R2 subunits (Figure 3B), and they interact with each other in a similar arrangement. Rigid body superpositions of individual R1 and R2 subunits (Figure 3B) or of R1 and R2 dimers, yield RMSD values of ∼3 Å. The pronounced concave surface present between the two R2 subunits is also present between the two R1 subunits. These surfaces pack against one another at a ∼45° angle in the tetramer, forming an X-shaped dimer of dimers (Figure 3C). In the tetramer, the closest distance between the C-terminus of an R1 subunit and the N-terminus of an R2 subunit—the gap that would need to be bridged by a peptide linker in a single-chain construct—is ∼50 Å (Figure 3D).

To investigate the relative contribution of the various subunit interfaces to the assembly of the holoenzyme tetramer, we used the online PISA server to conduct a structure-based analysis of the buried subunit interfaces throughout the complete tetramer. The results are shown in Supplementary Table S1. Out of the entire calculated buried interfacial surface area and corresponding free energy of association for the tetramer (5094 Å^2^; –37 kcal/mol), a substantial contribution is made by the R2–R2 interface (1838 Å^2^; –32 kcal/mol), whereas all other interfaces in the tetramer contribute ∼600–900 Å^2^ that are predicted to add a few extra kcal/mol of association energy. In particular, the R1–R2 interface that spans the putative DNA-binding cleft and two active sites is quite minimal in buried surface area and actually is predicted to be unstable on its own (+4 kcal/mol calculated free energy of association).

Since the R2 dimer is clearly the dominant assemblage in the BbvCI heterotetramer, a reasonable question is why an R2 homotetramer does not (or cannot) form. The answer to this question lies in the chemical and structural nature of the center of the heterotetrameric interface, which is comprised of four equivalent helices (two from the R1 subunits and two from the R2 subunits) that jointly contribute 12 charged residues (three from each helix) into a highly charged cluster of salt-bridges comprising 6 arginine residues and 6 glutamate and aspartate residues (the net charge in this region is zero) (Figure 3E). If we model a homotetramer of R2 subunits only (using the positions of the R1 subunits in the crystal structure as a guide), that cluster is disrupted, gaining a net positive charge and positioning four symmetry-related copies of Arg 199 into close contact with one another at the center of the tetramer (Figure 3F).

**Figure 4.**
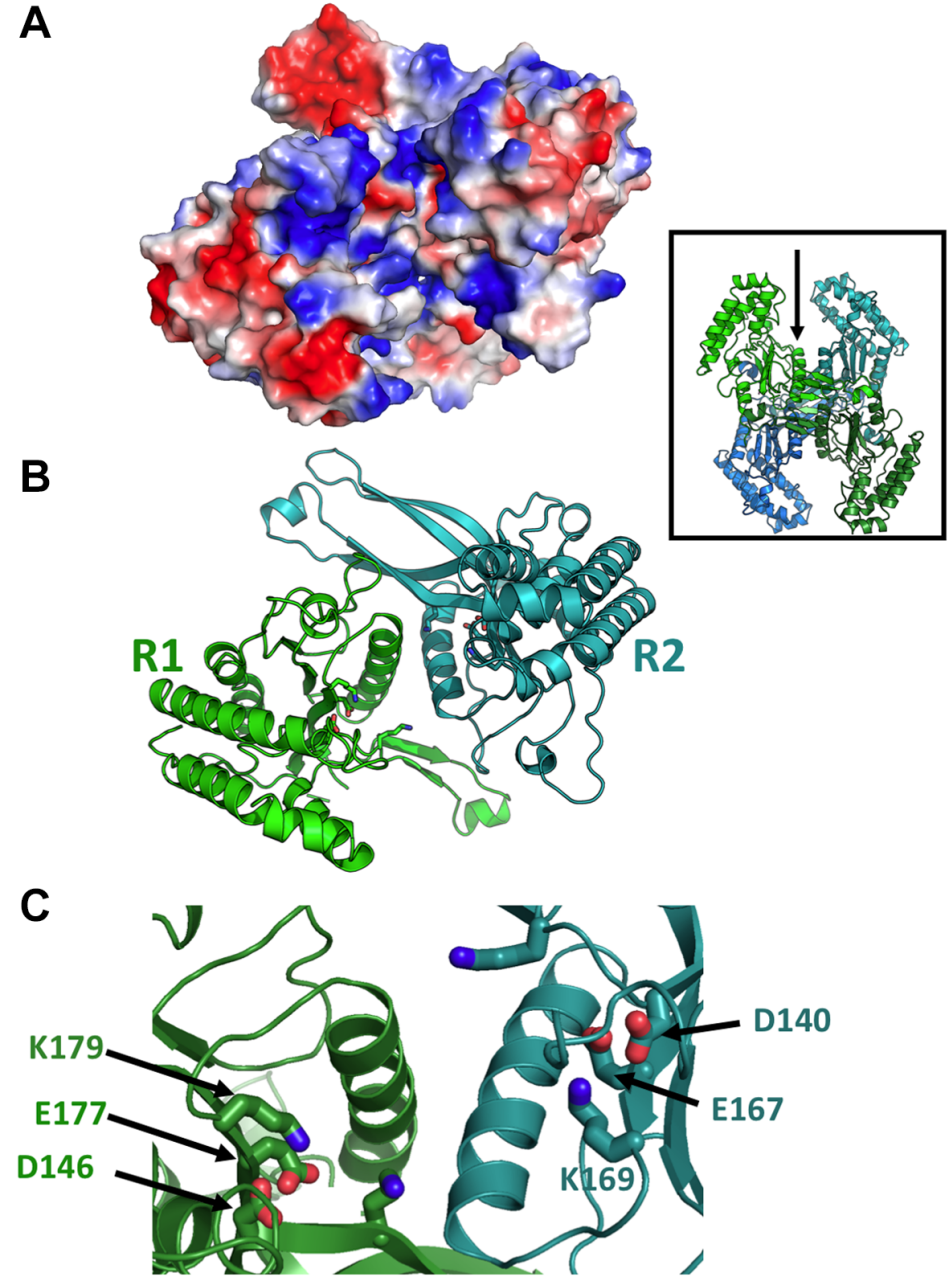
Proposed DNA-binding cleft and active site arrangement of an R1:R2 assemblage within the BbvCI heterotetrameric structure. **Panel A** (an electrostatic surface charge representation) and **Panel B**(ribbon diagrams) are shown in the same orientation, looking into the subunit interface and cleft (also illustrated with the arrow in the inset). The walls of the cleft are highly basic (blue color, representing overall positive charge) except for the two acidic residues of each catalytic site (red color, representing negative charge). **Panel C**displays a magnified image of the bottom of the protein interface and cleft, with the catalytic residues from each subunit labeled.

In the hetero-tetramer, two deep grooves are present on opposite sides of the molecule, each formed by residues from one R1 subunit, and one R2 subunit (Figures 3C and 4). The grooves are large enough to accommodate a DNA molecule, and display significant regions of positive surface charge on each side (Figure 4A). Within each groove, the catalytic site residues of one subunit are exposed on one side, and those of the other subunit are exposed diagonally across on the other side (Figure 4B and C). These paired sites occur ∼25 Å apart in opposing orientations, appropriately positioned to cleave double stranded DNA to produce a 3-base, 5′-overhang.

### The BbvCI holoenzyme undergoes dynamic subunit exchange

Although the R1 subunit dissociates from the holoenzyme at low pH and precipitates, both subunits with C-terminal 6xHis-tags remain soluble when expressed and purified individually at pH 8.0. These subunits were inactive on their own, but when mixed *in vitro* in approximately 1:1 stoichiometry, highly active enzyme was regenerated that was indistinguishable from wild type BbvCI (Supplementary Figure S6).

**Figure 5.**
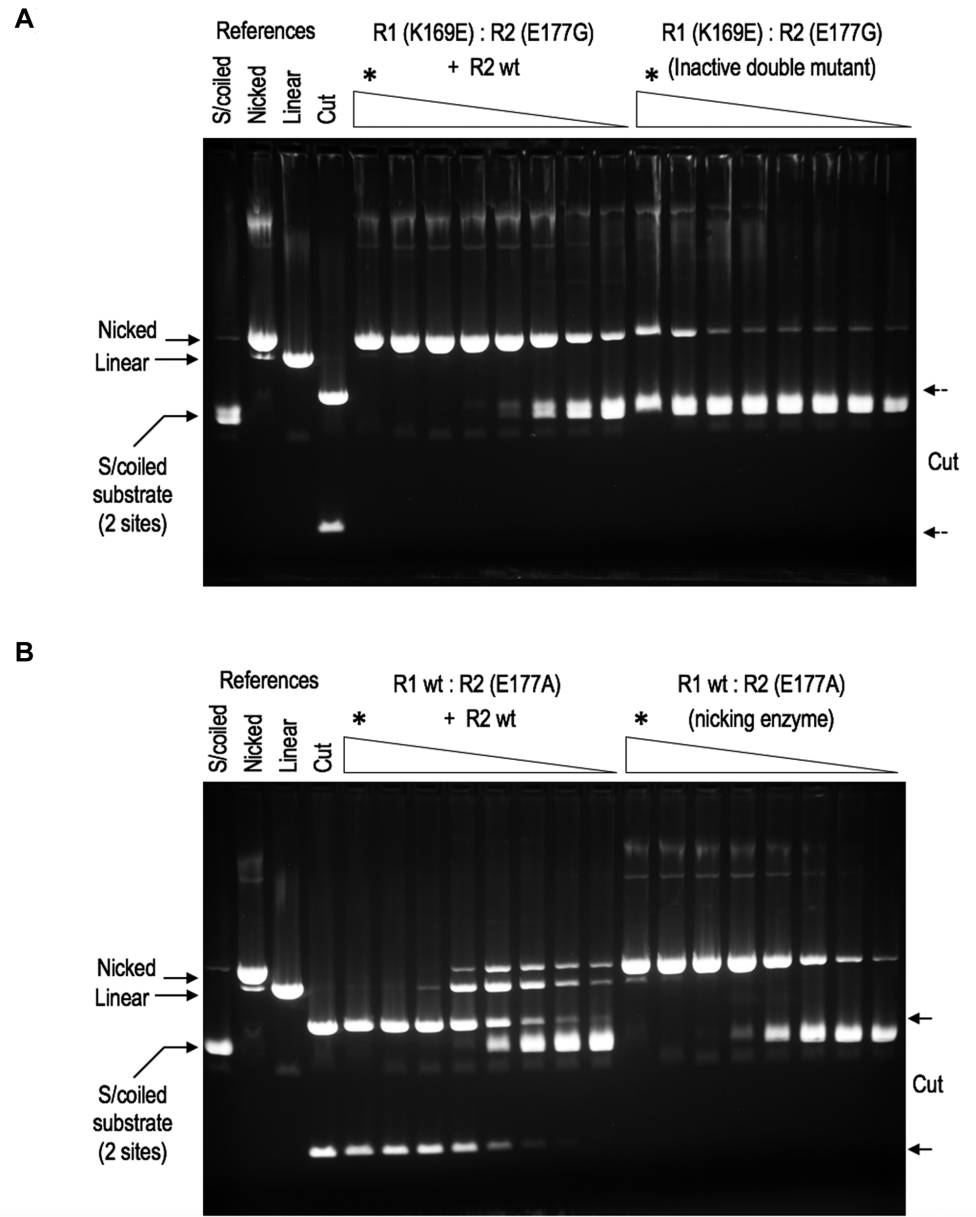
BbvCI subunit exchange. **Panel A:** An enzymatically inactive BbvCI variant, R1(K169E):R2(E177G), was constructed and partially purified. The protein was titrated in square-root 10 dilution steps on supercoiled plasmid DNA containing two target sites. Reactions were extracted with phenol/dichloromethane to remove DNA-bound protein, and analyzed by 1% agarose gel electrophoresis (upper panel, right). A trace of nicking activity is apparent at the highest enzyme concentrations (marked *), but no cleavage activity. The protein was mixed with the wild-type R2 subunit, assayed in the same way, and the mixture displayed approximately1,000-fold increase in DNA-nicking activity (upper panel, left). This new activity must stem from the *de novo* assembly of R1(K169E):R2 enzyme molecules that arise by *in vitro* subunit exchange. **Panel B:** A BbvCI nicking variant, R1:R2(E177A), was constructed and partially purified. The protein was titrated, extracted and electrophoresed as before (lower panel, right). Robust DNA-nicking activity is apparent with a trace of cleavage at the highest enzyme concentration (*). The protein was mixed with the wild-type R2 subunit, re-assayed and extracted in the same way, and the mixture displayed ∼10 000-fold increase in DNA-cleavage activity (lower panel, left). The new cleavage activity must stem from the *de novo* assembly of wild type R1:R2 enzyme molecules that again arise by *in vitro* subunit exchange. In each of these titrations, every tube contained 1 μg of substrate DNA ( = 1.1 pM target sites) and ∼3.16-fold (square-root ten) less protein than the preceding tube, such that every other tube contained 1/10 the amount. The titrations were initiated by adding the following quantities of protein to the first (leftmost) tube in each series, marked *: Panel A, right: 5.2 μg R1(K169E):R2(E177G) equal to 80 pM of each subunit. If 100% of these are present in tetramers, the stoichiometry in the first tube (*) is ∼35 inactive tetramers per target site. Panel A, left: 2.6 μg R1(K169E):R2(E177G) equal to 40 pM of each inactive subunit, plus 1.6 μg ( = 50 pM) active R2 subunit. At exchange equilibrium, these could assemble into ∼22 pM of catalytic nicking sites, for a stoichiometry in tube 1 of ∼20 catalytic sites per target site. Panel b, right: 2.2 μg R1:R2(E177A) equal to 34 pM of each subunit. If 100% of these subunits are present in tetramers, the stoichiometry in tube 1 is ∼60 catalytic sites per target site. Panel B, left: 0.17 μg R1:R2(E177A) equal to 26 pM of each subunit, plus 0.8 μg ( = 25 pM) R2 subunit. At equilibrium these could assemble into ∼12 pM of catalytic cleavage sites, for a stoichiometry in tube 1 of ∼10 catalytic sites per target site.

We exploited this behavior to further examine the holoenzyme's dynamic behavior. A double-defective variant of BbvCI was constructed in which both subunits carried a catalytically inactivating mutation, K169E in R1, and E177G in R2. This protein was enzymatically inert, but when mixed with the wild-type R2 subunit, which on its own is also inactive, robust single-strand nicking activity resulted (Figure 5A). This must stem from the *de novo* assembly of enzyme molecules containing the wild type R2 subunit in complex with the inactive R1 (K169E) subunit. Likewise, when the same wild-type R2 subunit was mixed with a bottom-strand nicking enzyme comprising wild-type R1 and mutant R2 (E177A) subunits, robust double-strand cleavage activity resulted (Figure 5B). Again, this must arise from subunit exchange and *de novo* assembly, in this case where both subunits in the complex are wild type. These results support previous observations that the holoenzyme exists in dynamic equilibrium with its components (46) and our current observation in this study of the labile nature of the R1–R2 complex.

### Cleavage of single-site and two-site DNA substrates by BbvCI

**Figure 6.**
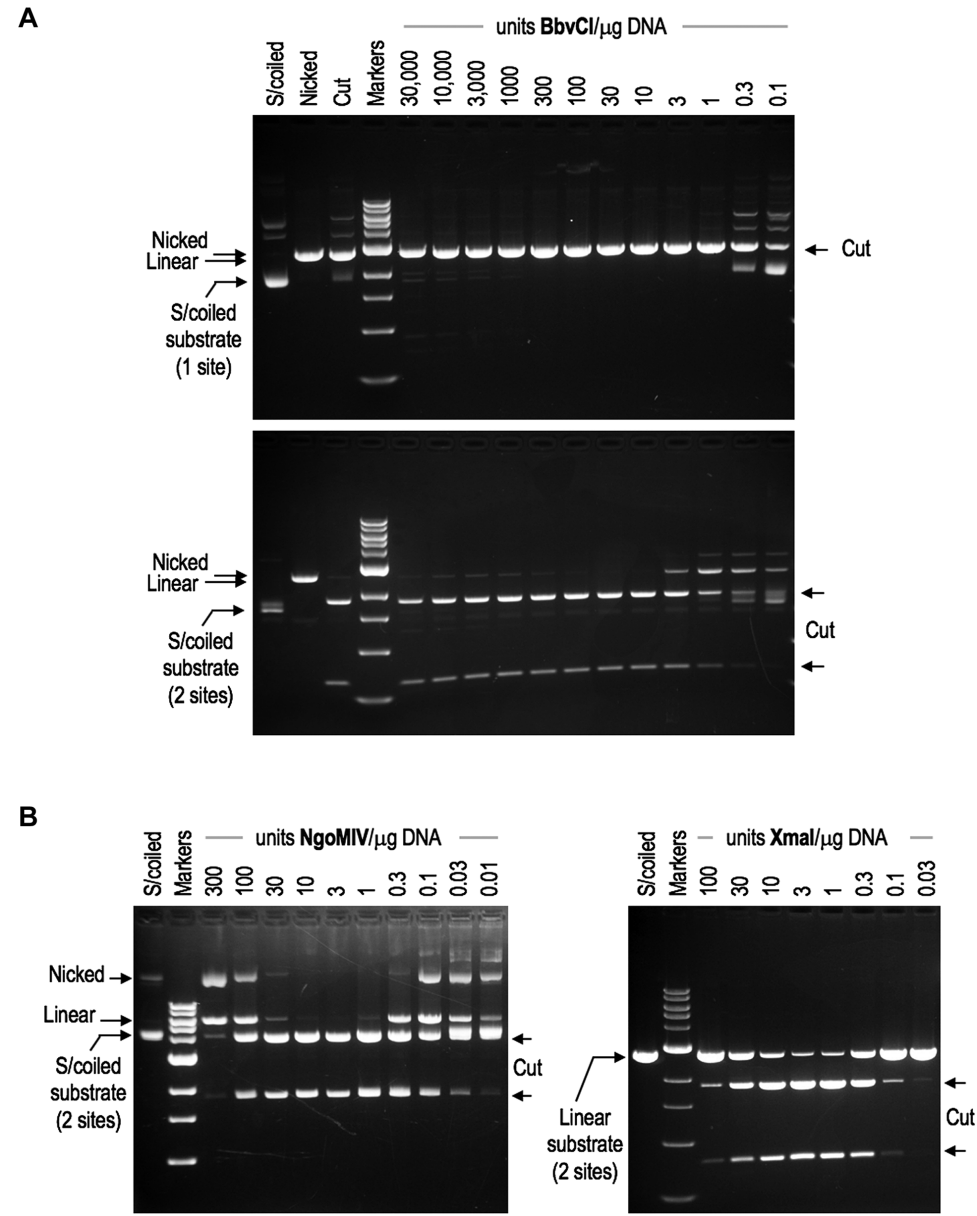
Cleavage behavior of wild-type BbvCI. **Panel A**: Purified BbvCI was titrated in square-root 10 (3.16-fold) dilution steps on supercoiled plasmid substrates containing one target site (upper panel) or two target sites (lower panel). Each tube contained 1 μg substrate DNA ( = 0.57 pM). 280 μg of BbvCI ( = 4.4 nM of each subunit) was added to the first tube in each series, a vast excess corresponding to ∼3900 tetramers per target site (upper panel) and ∼1900 per target site (lower panel). The proportion of active molecules in the enzyme preparation is not known, and so enzyme quantities are given in units of cleavage activity, one unit being the least amount required to completely digest one microgram of substrate DNA. On this scale, a ∼30 000-fold excess of active enzyme was added to the first tube of each series. Following incubation, reactions were extracted with phenol/dichloromethane to remove DNA-bound protein, and analyzed by 1% agarose gel electrophoresis. From these and similar titrations (not shown), it is evident that 1) BbvCI cleaves substrates with one site at least as rapidly as those with two sites; and, 2) cleavage is not suppressed at high enzyme concentrations, but continues to proceed to completion. These properties are characteristic of REases that cleave efficiently when bound to only one target sequence. **Panel B**: Cleavage behavior of NgoMIV and XmaI. Purified NgoMIV (left panel) and XmaI (right panel) were titrated on supercoiled (left) and linearized (right) plasmid substrates each containing two target sites. Excess enzyme was added to the first tube of each titration series and serially diluted in square-root 10 steps. Following incubation, reactions were extracted and analyzed by 1% agarose gel electrophoresis. In contrast to BbvCI, cleavage by NgoMIV and XmaI is noticeably suppressed at high enzyme concentrations, and no longer proceeds to completion. This behavior is characteristic of REases that must bind two target sites at once in order to cleave, and is a property of many that act as tetramers.

Many tetrameric REases cleave DNA only when bound to two target sites at once (26,64,65). Typically, these enzymes cleave substrates with two sites faster and more completely than substrates with one site (36,66,67). In addition, their ability to cleave declines significantly at high enzyme concentrations, as few enzyme molecules can find two unoccupied sites to bind at once. Despite its tetrameric crystal structure, BbvCI does not behave in this way. BbvCI cleaves plasmid substrates with one site at least as efficiently as those with two sites (Figure 6A), and cleavage efficiency is unaffected by enzyme to target-site stoichiometry. Tetrameric REases typically begin to cleave poorly above a 5-fold stoichiometric excess—equivalent to ∼100-fold over-digestion (Figure 6B). BbvCI, by contrast, cleaves to completion at even >1000-fold stoichiometric excess (Figure 6A). In these respects, BbvCI behaves more like an orthodox dimeric Type IIP REase such as EcoRI or BamHI, than it does a typical tetramer.

### Tethered, single-chain BbvCI enzyme constructs

**Figure 7.**
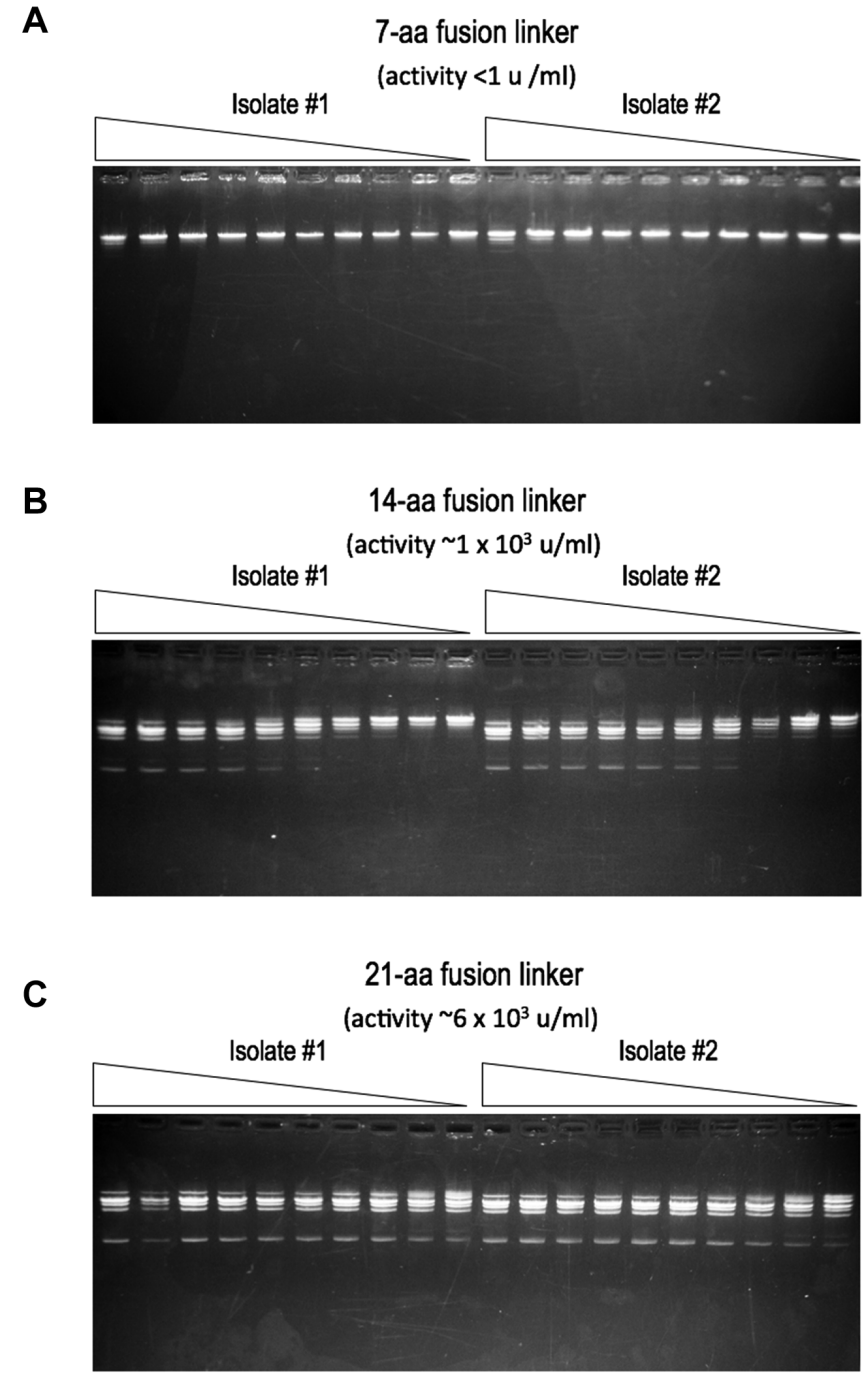
Cleavage activities of tethered, single-chain variants of BbvCI. The wild-type R1 and R2 genes were joined in-frame with oligonucleotide linkers that introduced 7, 14, or 21 additional codons between the two genes (**Panels A, B** and **C**,respectively). Two independent isolates of each construct were sequence-verified, and the fused protein each encoded was partially purified and titrated in 2-fold dilution steps on linear phage lambda DNA. Reactions were extracted with phenol/dichloromethane and analyzed by 1% agarose gel electrophoresis. The construct with the longest linker (panel C) displayed highest activity (∼6 × 10^3^ units/ml of extract), corresponding to a specific activity of ∼4 × 10^6^ units per mg of protein, or approximately the same as the two-subunit parental enzyme. The construct with the intermediate linker (panel B) displayed ∼15% of this activity (∼1 × 10^3^ units/ml extract), and the construct with the shortest linker (panel a) was essentially inactive (<1 unit/ml).

To further validate the organization of the tetrameric assemblage of the BbvCI holoenzyme described above, we tethered the R1 and R2 subunits together into a single polypeptide chain. Three different linking oligonucleotides, 7, 14 and 21 codons in length, were used to connect the 3′ terminal codon of the R1 gene to the 5′ initiation codon of the R2 gene. The resulting protein constructs (see inset of Figure 3 for the relative location of the two conjoined termini) were purified in duplicate and assayed for cleavage activity. The construct with the 21-aa linker had an estimated specific activity of 4 × 10^6^ units per mg of protein—similar to that of the parental wild-type enzyme (∼1 × 10^6^ units per mg). The construct with the 14-aa linker displayed ∼15% of this activity, and the construct with the 7-aa linker was inactive (Figure 7). These results agree with the subunit organization of the tetramer observed in the crystallographic analysis described above: the shortest distance that a tether would need to span from the C-terminus of an R1 subunit to the N-terminus of an R2 subunit is ∼50 Å, necessitating a linker of at least 14, and preferably more, residues to allow proper assembly of the tetramer.

### Structural similarity analyses and computational modeling of BbvCI bound to DNA

Despite considerable effort, we were unable to generate co-crystals of the enzyme bound to its DNA target site. In the absence of such a structure, we resorted to structure-based modeling to create a sterically reasonable model of the complex that might facilitate investigation of the protein–DNA recognition mechanism.

**Figure 8.**
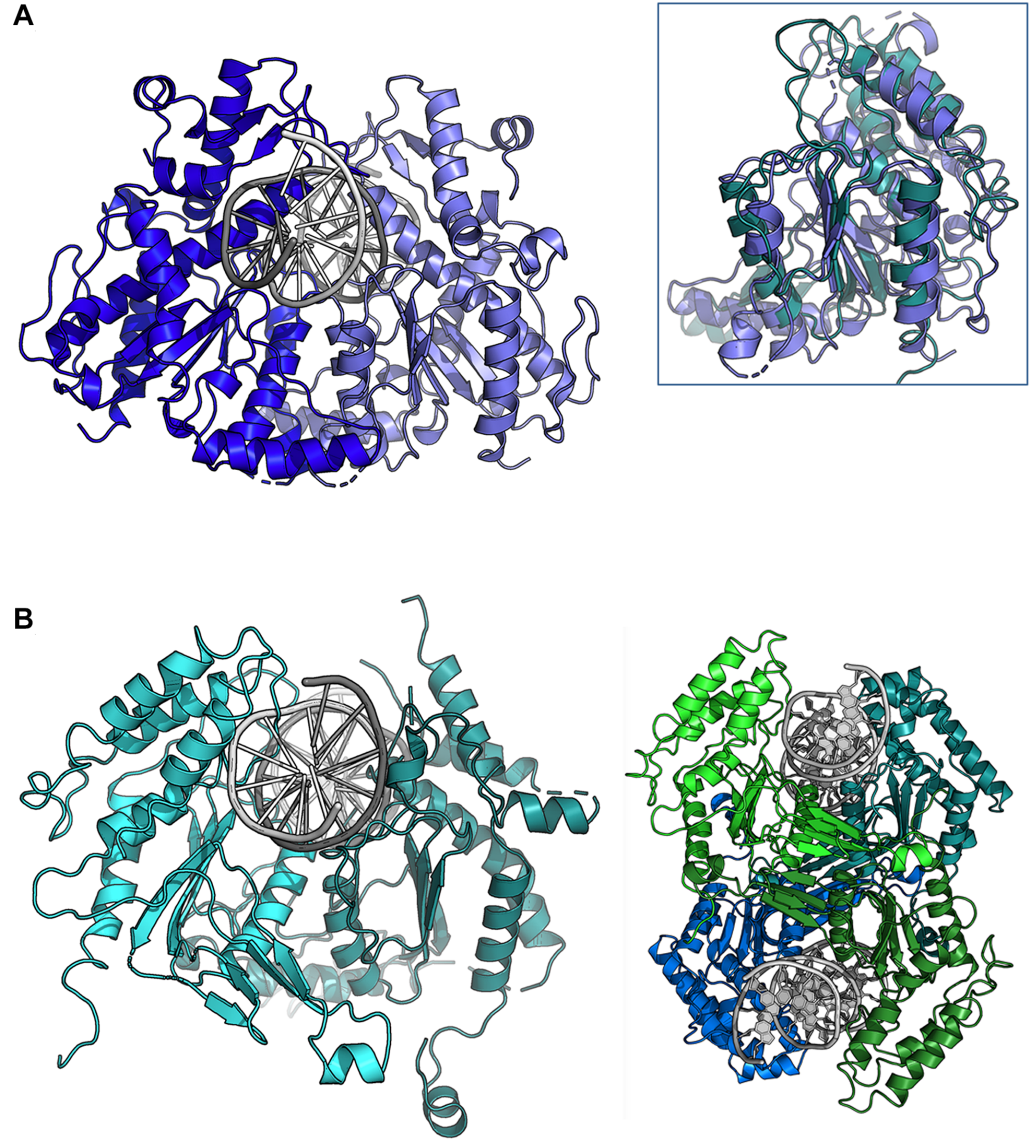
Computational model of BbvCI-DNA complex. **Inset**:Superposition of SgrAI and BbvCI subunits. **Panel A**: Crystal structure of the SgrAI-DNA complex. **Panel B**:Docked model of a BbvCI-DNA complex. To the right is the same model shown with the entire tetramer (same relative orientation as in Figure 3) with two corresponding docked DNA duplexes.

Submission of the coordinates of the BbvCI R2 subunit to the DALI and FATCAT structural similarity servers (68,69) indicated that the closest related structures in the protein database corresponded to the SgrAI/NgoMIV family of Type II restriction endonucleases, the structures of several of which have been determined bound to their DNA targets. The servers also produced alignments of individual subunits from BbvCI and SgrAI corresponding to an RMSD of ∼3.0 Å (*P*-value = 0.0074) across 196 equivalent positions (Figure 8, inset).

SgrAI acts in multiple oligomeric forms, and cleaves a symmetric 8 base pair target site (5′ - CR/CCGGYG - 3′, and its reverse complement 5′- GY/GGCCRC-3′) to generate 4-base, 5′-overhangs. It recognizes a target site of similar length to BbvCI, and produces product ends that differ in length by only one base relative to BbvCI, cleaving phosphodiester bonds on each strand that are separated by four base pairs, rather than three. In the structure of the SgrAI homodimer bound to its target site (62), the two protein subunits interact with each other much as the R1 and R2 subunits interact in the BbvCI holoenzyme, but the cleft between them is occupied by the bound DNA duplex, and the two SgrAI subunits are rotated slightly towards one another to close around the DNA.

Relying upon: (i) the structural similarities between SgrAI and BbvCI, (ii) the availability of a high-quality DNA-bound structure of SgrAI and (iii) the well-established cleavage specificity and orientation of BbvCI on its DNA target (which constrains the catalytic residues of each subunit to be in close proximity of a defined phosphate in each DNA strand), we generated an initial docked representation of DNA-bound BbvCI and then used the *Rosetta* protein design program suite to produce an energy minimized model of the complex (Figure 8, panel b).

### DNA sequence recognition by BbvCI

**Figure 9.**
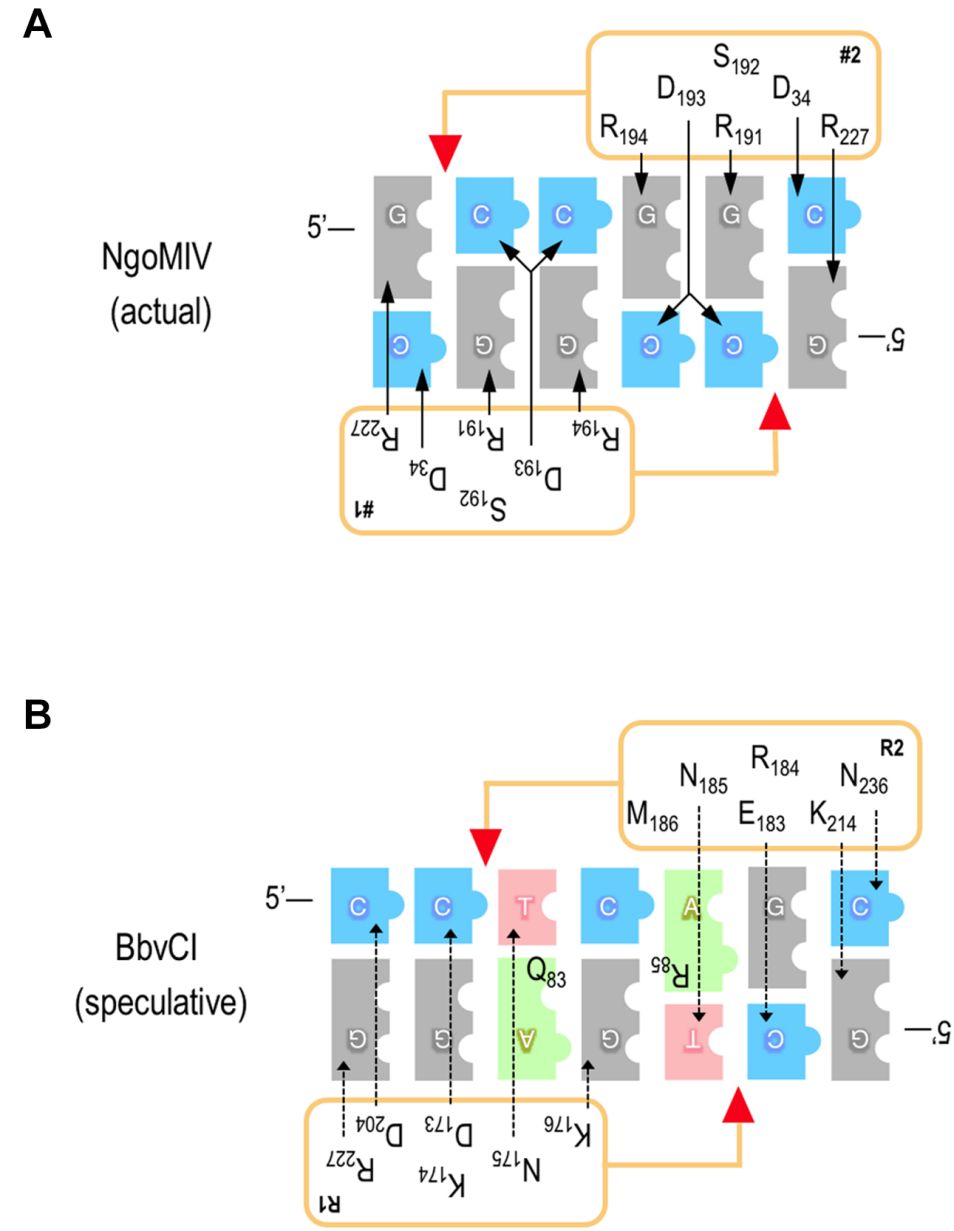
Observed and proposed amino acid-base pair interaction schematics for NgoMIV and BbvCI. **Panel A**: The NgoMIV interactions (upper panel) are inferred from the protein–DNA co-crystal structure (26), and mirror those of the related enzymes, SgrAI and Bse634I (62,70). **Panel B**:The BbvCI interactions (lower panel) are speculative, and derive from aa sequence and structure alignments. The bases of the target sequences are shown as blue (cytosine), grey (guanine), red (thymine), and green (adenine) blocks. Protein subunits are depicted as rectangles alongside the DNA; red triangles indicate the cleavage positions of the catalytic sites. Amino acids inferred or speculated to determine sequence-specificity are listed within the rectangles. These are the same in the two NgoMIV subunits (labeled #1 and #2) because the subunits are identical, but they are different in the two BbvCI subunits (labeled R1 and R2) because these differ. Arrows connect amino acids to the base(s) they specify, and imply the presence of one or more hydrogen bonds. Solid arrows signify confidence in the interactions of NgoMIV. Dotted arrows signify uncertainty in the interactions of BbvCI, and those shown depict only one of several possibilities.

Since we could not crystallize BbvCI bound to its DNA target, we could not observe directly the interactions between protein residues and DNA base pairs that facilitate recognition of the CCTCAGC target sequence. However comparisons with related REases, and the modeling exercise described above, led us to conclude that residues 173–176 of the R1 subunit, and 183–186 of the R2 subunit, likely participate in recognition of the inner five bp of the target sequence (-CTCAG-) (Figure 9; described further in the Discussion), and in particular that R1 Lys176 and R2 Met186 recognize the central C:G bp of this sequence.

**Figure 10.**
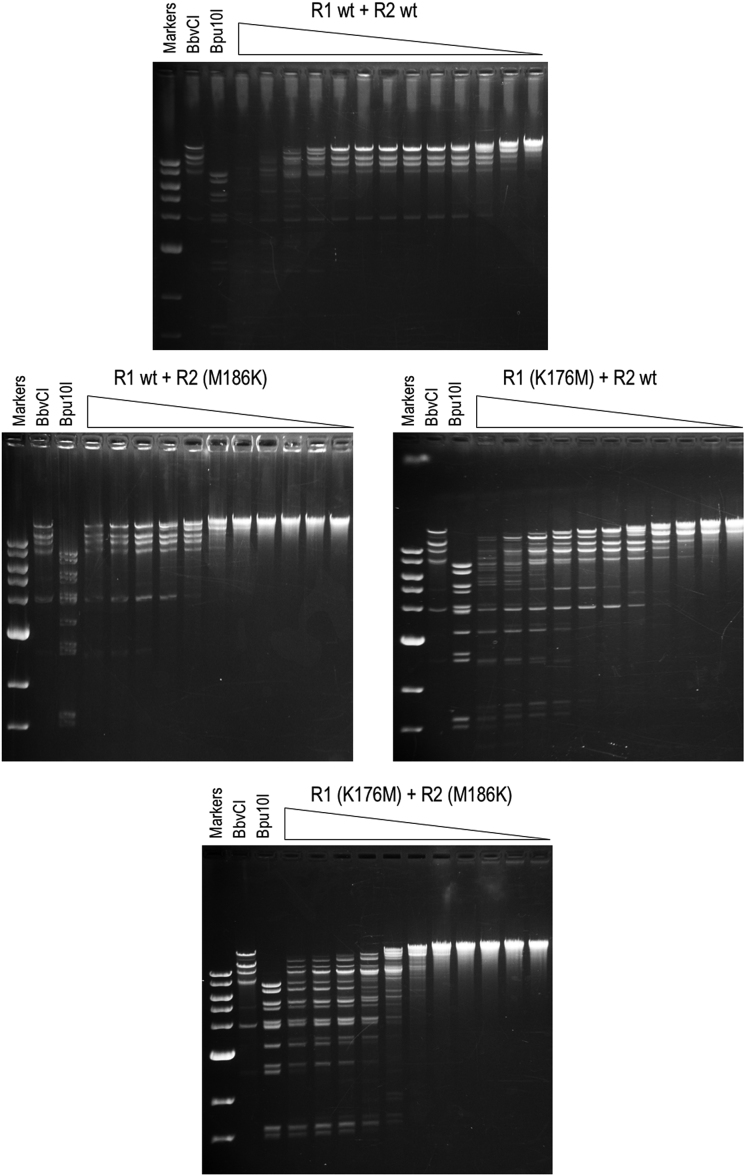
Cleavage properties of BbvCI specificity mutants. C-terminal His-tagged forms of the R1 and R2 wild type subunits, and of the R1 (K176M) and R2 (M186K) mutant subunits, were purified individually to near-homogeneity, as determined by SDS-PAGE. Each subunit was assayed alone for endonucleolytic activity by titration on supercoiled plasmid DNA (two BbvCI sites), starting with at least a 50-fold subunit:plasmid stoichiometric excess. No DNA-nicking or DNA-cleavage activity was detected (not shown). Equal quantities of subunits were then mixed and assayed by titration (Cutsmart^®^ buffer, 37°C, overnight) in square-root 10 dilution steps on phage lambda DNA (7 BbvCI sites; 19 Bpu10I sites) followed by agarose-gel electrophoresis. Each titration tube contained one microgram DNA (= 0.032 pMole). Top panel, first tube: 37 nM R1 + 37 nM R2; starting stoichiometry (i.e. number of molecules of each BbvCI subunit per lambda DNA molecule in tube 1) = 1150:1. Middle panel left, first tube: 30 nM R1 + 30 nM R2(M186K); starting stoichiometry = 950:1. Middle panel right, first tube: 26 nM R1 (K176M) + 26 nM R2; starting stoichiometry = 820:1. Bottom panel: 23 nM R1(K176M) + 23 nM R2(M186K); starting stoichiometry = 710:1. Note that the fragmentation pattern in the top panel matches that of stock BbvCI, signifying cleavage at CCTCAGC, while the fragmentation pattern in the bottom panel closely matches that of Bpu10I, signifying cleavage at CCTNAGC due to loss of specificity for the central base pair in the recognition sequence. (In all but the lower panel, extended overnight digestion coupled with the vast initial enzyme:substrate excess, has led to non-specific degradation of the substrate in the first few lanes of the titrations.)

To test this latter idea, we mutated R1 K176 and R2 M186, purified the resulting subunits, and examined the cleavage properties of various 1:1 subunit mixtures (Figure 10). As expected, the control mixture of wild type R1 and wild type R2 generated the regular BbvCI digestion pattern, indicative of cleavage at only CC|TCAGC. R1 wild type mixed with the R2 (M186K) mutant did much the same, albeit at 10^3^-fold lower specific activity. R2 wild type mixed with the R1 (K176M) mutant generated the regular BbvCI pattern at low enzyme concentrations, but at higher concentrations cleaved at many additional sites. A mixture of both mutant subunits, R1 (K176M) plus R2 (M186K), generated at all concentrations a pattern almost identical to that of Bpu10I, which cleaves CC|TNAGC (Figure 10 and Table 3). Mutating both R1 Lys176 and R2 Met186 abolishes the specificity of BbvCI for the central bp of its target sequence, then, resulting in a less discriminating enzyme that now accepts any bp at this position instead of only the C:G bp tolerated by the wild-type enzyme.

**Table 3.** Cleavage behavior of BbvCI mutants, summarized from Figure 10. Column 1: wild type and mutant subunits were purified individually and assayed for endonucleolytic activity alone or in 1:1 mixtures. Column 2: Individual subunits displayed no detectable DNA-nicking or DNA-cleavage activity (rows 1–4); mixtures displayed BbvCI-like cleavage behavior (rows 5 and 6), Bpu10I-like cleavage behavior (row 8), or intermediate behavior (row 7). Column 3: mixtures involving mutant subunits (rows 6–8) exhibited lower specific cleavage activity than the wild-type mixture (row 5)

Subunit composition	Cleavage behavior	Relative activity (%)
R1 wt	None	0
R2 wt	None	0
R1 (K176M)	None	0
R2 (M186K)	None	0
R1 wt + R2 wt	CCTCAGC	100
R1 wt + R2 (M186K)	CCTCAGC	0.1
R1 (K176M) + R2 wt	CCTCAGC >>CCTNAGC	0.4
R1 (K176M) + R2 (M186K)	CCTNAGC	0.1

## DISCUSSION

Despite only ∼27% aa sequence identity, the structures of the two BbvCI subunits are quite similar. Analysis of the more precisely modeled R2 subunit using the Vector Alignment Search Tool (VAST) at NCBI revealed close structural matches to NgoMIV and related enzymes, several of which have been crystallized bound to their DNA targets (26) (62,70). The similarities are especially close at the protein–DNA interfaces formed by the catalytic and specificity residues, and allow us to tentatively infer the amino acids of BbvCI involved in DNA sequence-recognition.

In Type II REases of these kinds, the subunit that cleaves the ‘bottom’ DNA strand primarily specifies the left half of the target sequence, as conventionally written, and the subunit that cleaves the ‘top’ strand primarily specifies the right half (Figure 9). Extrapolating this to BbvCI, we expect the R1 subunit to specify mainly the left side of the target sequence, 5′-CCTN or 5′-CCTC, and the R2 subunit to specify mainly the right side, 5′-GCTN or 5′-GCTG. (Sequence recognition by REases usually involves contacts to both bases of each base pair, but following convention, only the 5′-to-3′ strands are shown here.)

NgoMIV recognizes the symmetric sequence G|CCGGC, and each of its identical subunits specifies 5′-GCC (26). The inner two C:G base pairs of these half-sites are contacted in the major DNA groove by a loop-helix motif, R-S-D-R (Arg191-Ser192-Asp193-Arg194) 3-aa away from K187, the terminal lysine of the PD-(D/E)XK catalytic site (Figure 9A). A similar arrangement occurs in the closely related REases SgrAI, Cfr10I and Bse634I (62,70,71). The equivalent residues of BbvCI are D-K-N-K (Asp173-Lys174-Asn175-Lys176) in R1, and E-R-N-M (Glu183-Arg184-Asn185-Met186) in R2. Given the close structural similarities in this part of the proteins, we expect that these residues likewise make major-groove contacts to the inner base pairs of the BbvCI half-sites. There are numerous ways in which this could occur (72), and one such possibility is shown in Figure 9B. The 7-bp recognition sequence of BbvCI is one bp longer than that of NgoMIV. This difference likely stems from the dimerization interfaces, which position the BbvCI subunits slightly further apart than those of NgoMIV, and in so doing reduce the 4-base cleavage offset of NgoMIV to 3 bases in BbvCI.

We demonstrate above that Lys176 of the R1 subunit is the major determinant of the central C:G bp of the recognition sequence. We speculate that Lys176 specifies the G of this base pair, in part by donating H-bonds to the guanine *O*6 and/or N7 atoms, and that the corresponding, symmetrically opposed, Met186 of the R2 subunit juxtaposes the cytosine (Figure 9B). Due to its limited capacity to form H-bonds, methionine plays a more passive role in sequence-recognition than lysine. Consistent with this interpretation, we find that Bsu36I (CC|TNAGG), BlpI (GC|TNAGC), and Bpu10I (CC|TNAGC), REases related to BbvCI that do not specify the central bp, all have methionine at this position (Supplementary Figure S7).

In proteins with very high fidelity of sequence-recognition such as REases, discrimination of A:T and T:A base pairs is often augmented by a residue in the minor DNA groove that obstructs the 2-amino group of guanine, preventing G:C and C:G bp from fitting there (unpublished observations). Modeling suggests that both BbvCI subunits have minor groove residues that might act in this way at the T:A bp in each half-site, namely Arg85 (or Trp84) of R1 and Gln83 (or Glu84) of R2. Arg and Gln also occur at the equivalent positions in the Bsu36I, BlpI, and Bpu10I subunits which specify T:A at the same position in their half-sites (Supplementary Figure S7).

The outer G:C bp of each NgoMIV half-site is specified by Arg227 and Asp34, amino acids distant from each other in the linear protein sequence, and distant from the R-S-D-R motif that recognizes the inner base pairs (26). Structural similarities with the BbvCI subunits are poor in these regions, complicating identification of the residues that might specify the outer base pairs of the BbvCI half-sites. Notwithstanding, structural superimposition and sequence co-variation suggest that Asp204 and Arg227 of the R1 subunit might together specify the outer C:G of the left half-site (CCTC), and Lys214 and Asn236 of the R2 subunit might together specify the outer G:C of the right half-site (Figure 9B).

Most Type II restriction endonucleases act as homo-multimers composed of two or more copies of the same protein subunit. Because such enzymes are symmetric, they recognize DNA sequences that are also symmetric, and they cleave the two strands symmetrically at equivalent positions. Type II REases composed of different subunits—hetero-multimers— are relatively rare. And those like BbvCI, whose subunits are similar in size and sequence, are even more rare. It is tempting to speculate that the subunits of BbvCI share common ancestry, and descend from symmetric enzymes that recognized slightly different sequences. Candidates for such predecessors can found in Bsu36I (CC|TNAGG), whose subunits recognize the left half of the BbvCI sequence, BlpI (GC|TNAGC), whose subunits recognize the right half, and Bpu10I, a chimaera of the two that recognizes the hybrid sequence, CC|TNAGC. As shown above, a change of only one or two amino acids could be sufficient to change this specificity to that of BbvCI:CC|TCAGC. That the subunits of these four enzymes have similar sizes, cleave at the same position, and share significant aa sequence similarity lends support to this idea, as too, perhaps, does the observation that the BbvCI subunits attach to each other rather weakly.

## DATA AVAILABILITY

The data and coordinates for all crystal structures described in this paper have been deposited at www.rcsb.org. ID codes for the individual data sets and coordinates are provided in Table 2.

## Supplementary Material

Supplementary Data
