# Supplementary material for: Structure, subunit organization and behavior of the asymmetric Type IIT restriction endonuclease BbvCI
Source: Nucleic Acids Res. 2018 Nov 5;47(1):450–67. doi: 10.1093/nar/gky1059 (PMC6326814; doi:10.1093/nar/gky1059)

**Supplementary Information for “Structure, subunit organization and behavior of the asymmetric Type IIT restriction endonuclease BbvCI” (Shen et al.)**

**Supplementary Figure S1.** SDS-PAGE visualization of purified wild-type BbvCI used for crystallization trials. The R1 subunit (lower band) is 10-aa shorter than the R2 subunit (upper band), and migrates slightly faster. The two subunits are present in equimolar (1:1) proportion.

**
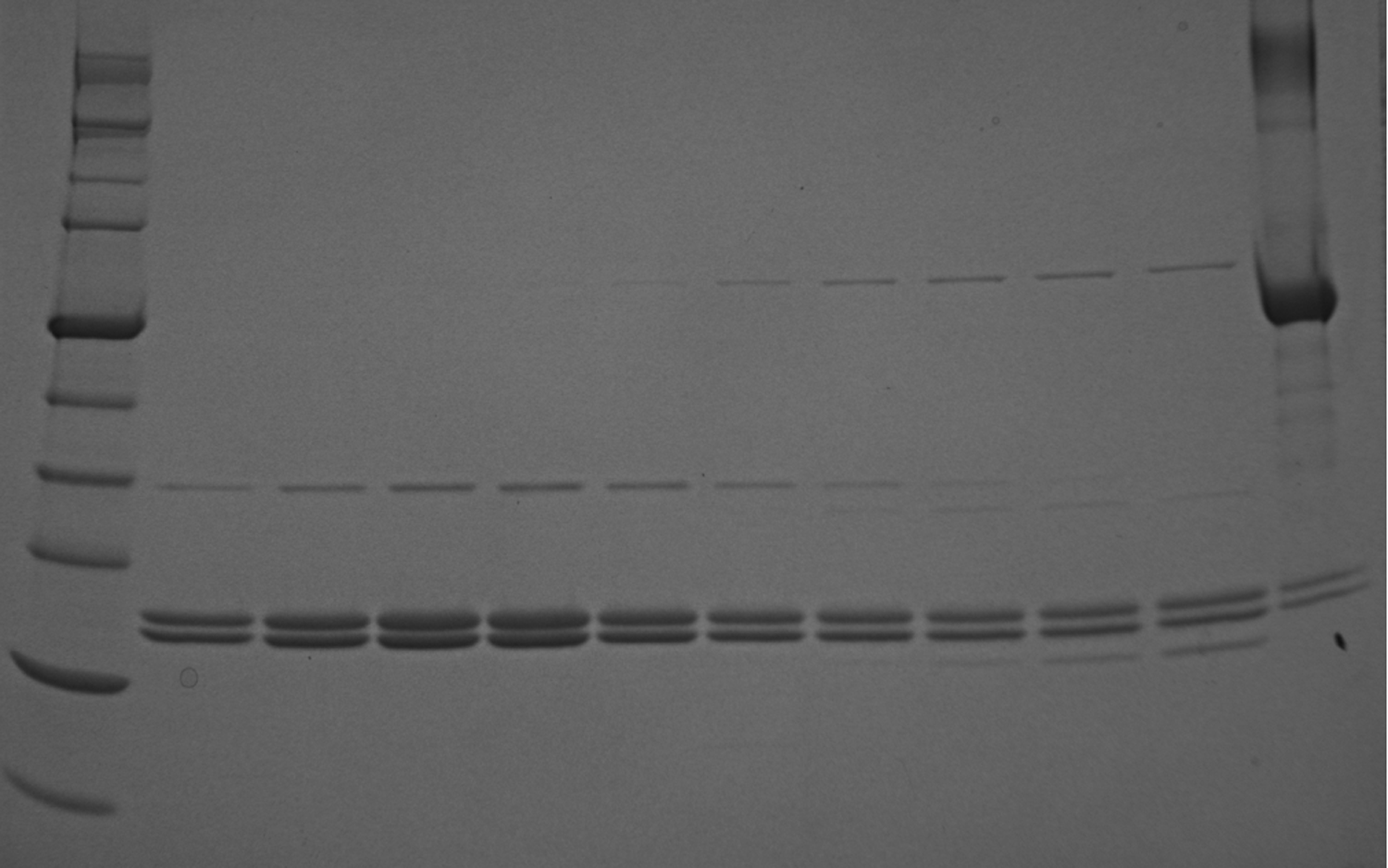
**

**Supplementary Figure S2.** Initial electron density produced by multiple isomorphous replacement (MIR) phase calculations (blue), showing the electron density corresponding to clusters of 3 iodine atoms in the 5-Amino-2,4,6-triiodoisophthalic acid (’I3C’) heavy atoms derivative (green spheres).


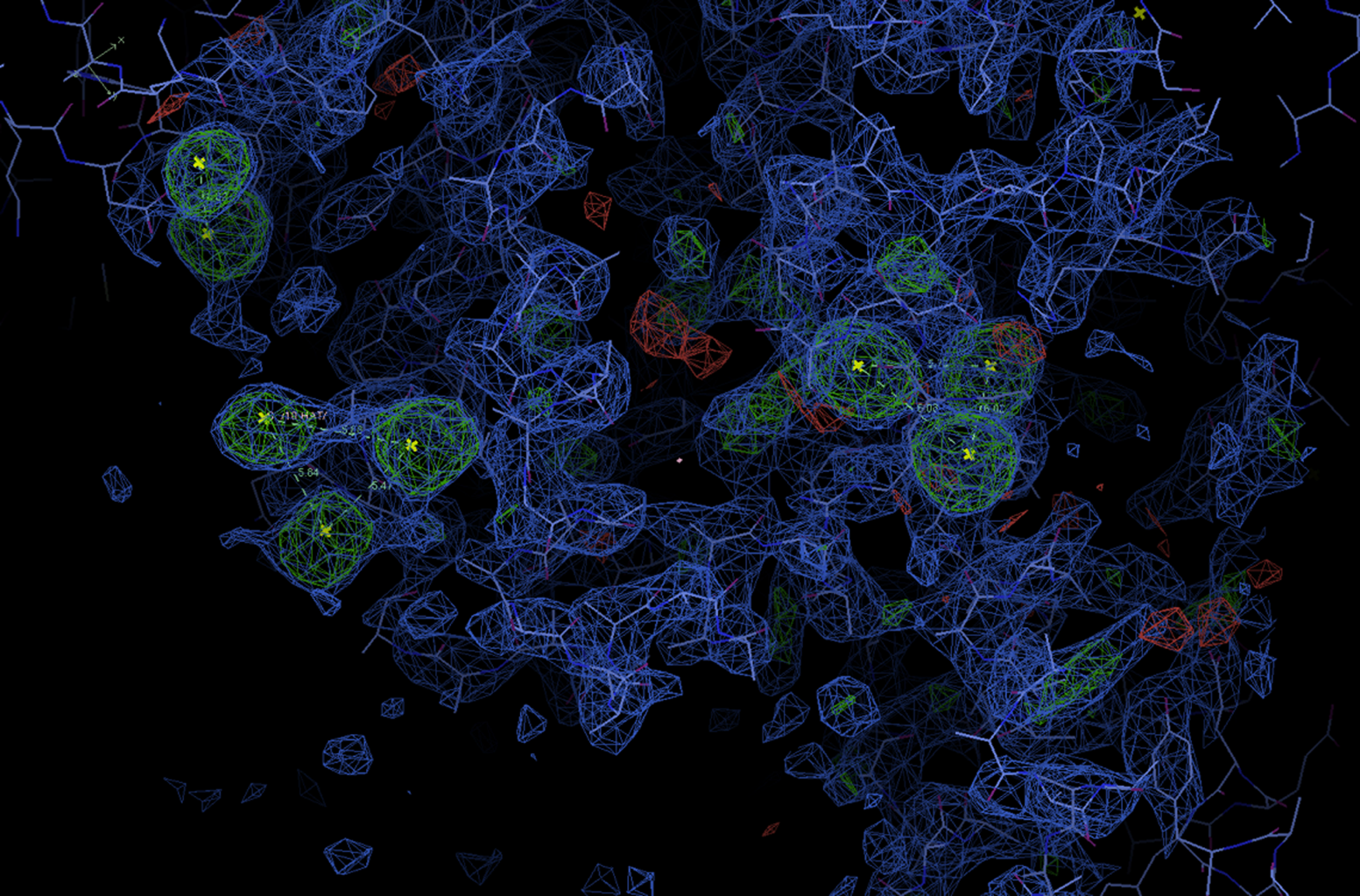


**Supplementary Figure S3. Catalytic activities of R1^+^ : R2^—^ point mutants.** Selected amino acids in the R2 subunit were changed to alanine by site-specific mutagenesis. These were paired with the wild type R1 subunit, and the resulting enzymes were partially purified, incubated with supercoiled plasmid DNA (two BbvCI sites), and analyzed by 1% agarose gel electrophoresis. Some mutations abolished activity (e.g. E211); others had little effect (e.g E163); and those in catalytic residues inactivated the catalytic site resulting in ’DNA-nicking’ enzymes (e.g. D146). These results indicate that D146 forms the proximal part of the PD-(D/E)XK motif of the R2 subunit, and E177 and K179 form the distal part. These and other results are summarized in Table 1.


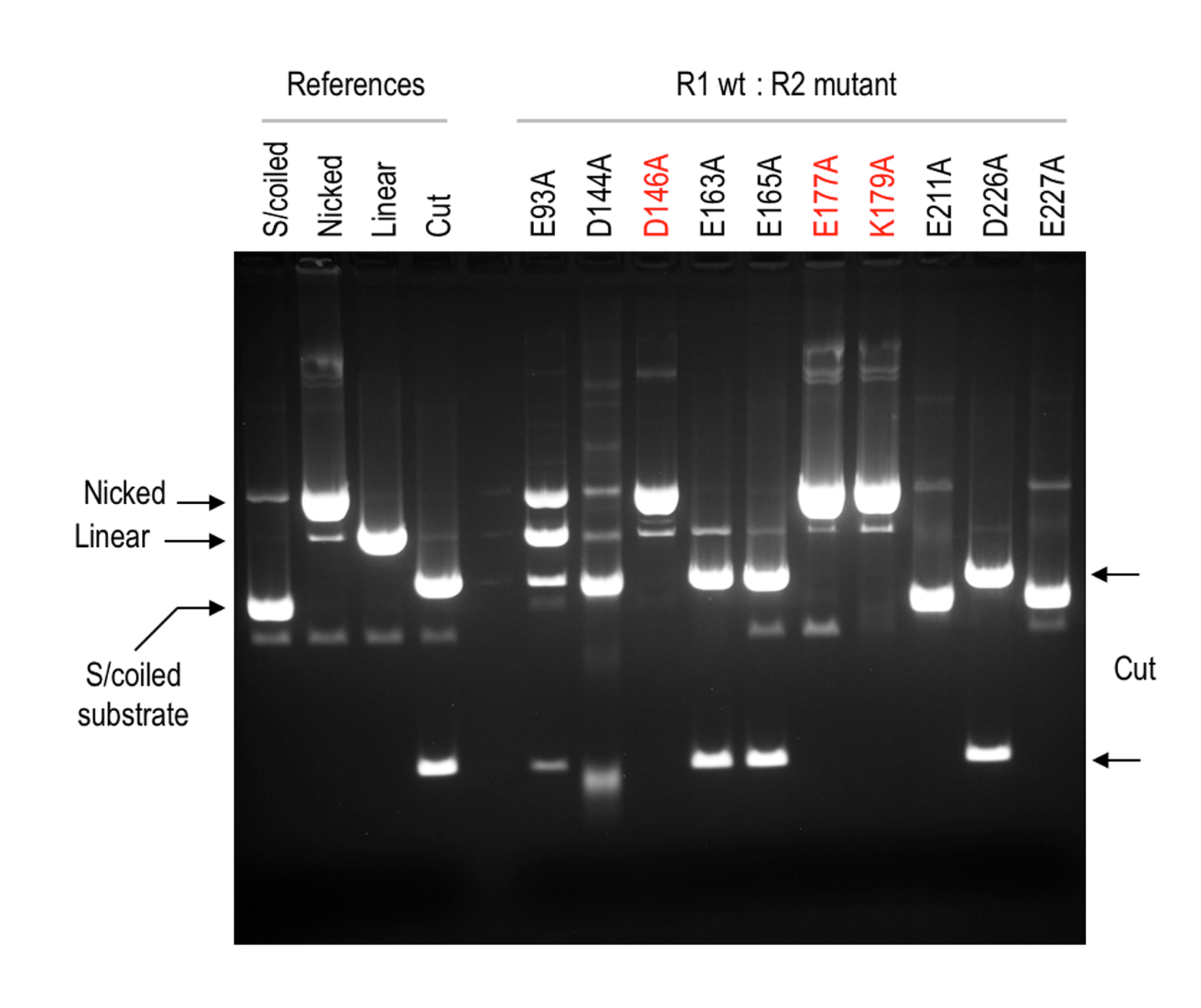


**Supplementary Figure S4**. **Size exclusion chromatographic profiles of BbvCI using buffers corresponding to pH 7.5 and 4.5.** The experiments were conducted using a Superdex 200 (10/300) sizing column with a flow rate of 0.5 mL/min. Buffer conditions were 20 mM HEPES pH 7.5, 150 mM NaCl and 20 mM sodium acetate pH 4.5, 150 mM NaCl, respectively.

**
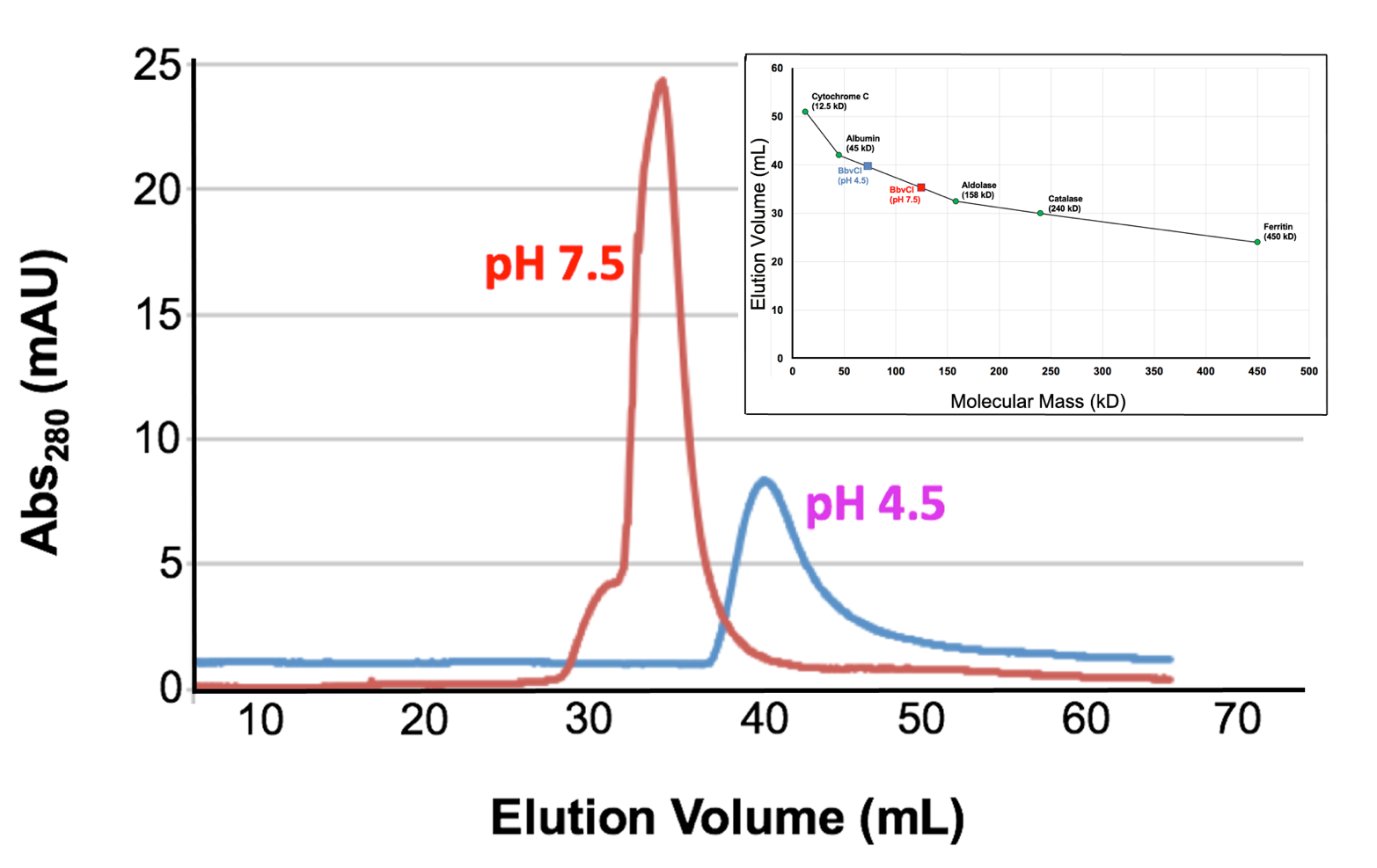
**

**Supplementary Figure S5. Representative electron density map of the BbvCI tetramer.** The map is a region of unbiased density from the initial molecular replacement solution, and corresponds to the central region of the molecule illustrated further in **Figure 3.**

**
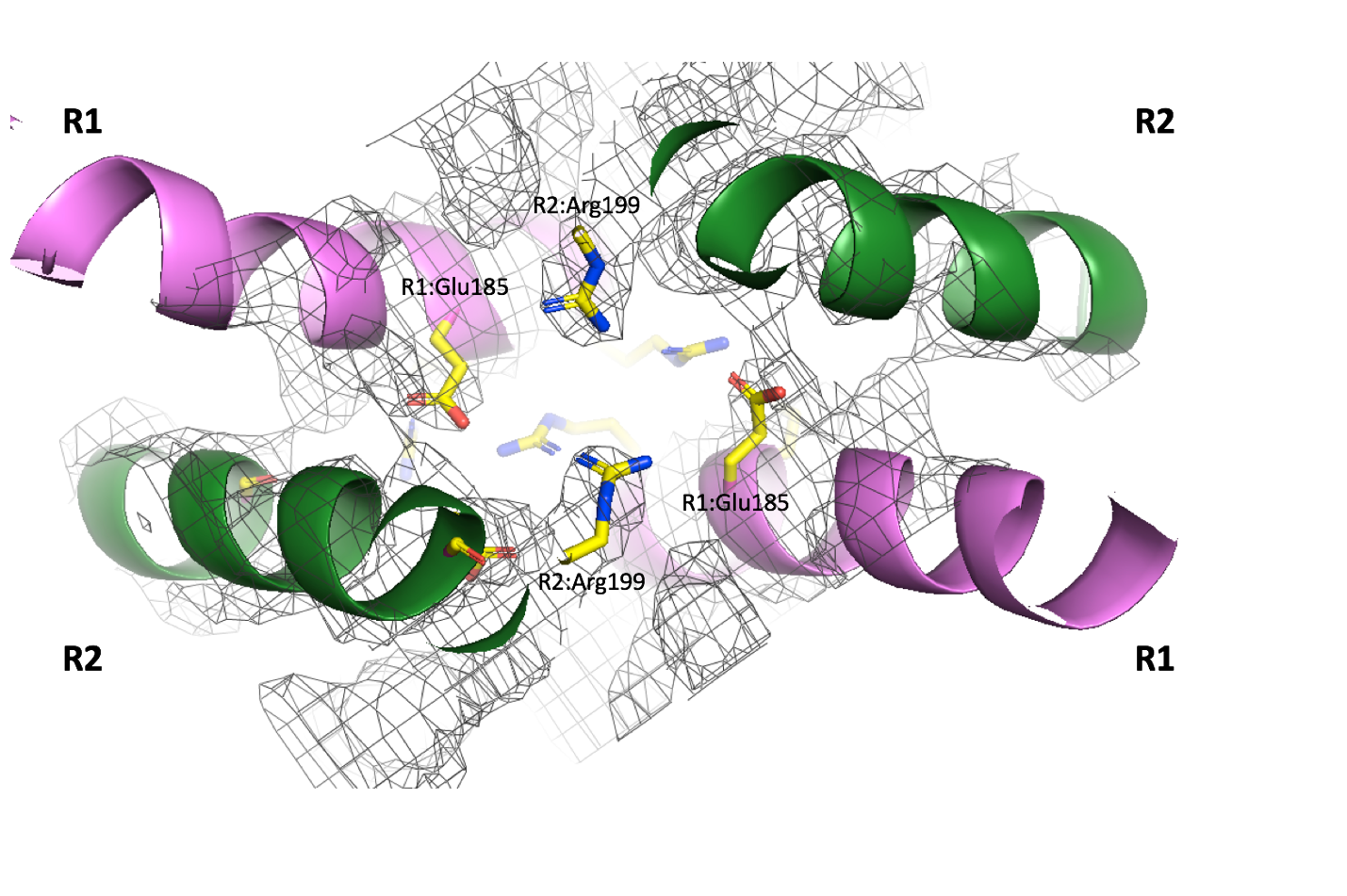
**

**Supplementary Figure S6. Re-association of independently purified BbvCI subunits. *Panel a:*** His-tagged forms of the individual wild type BbvCI R1 and R2 proteins were partially purified. Each was assayed for enzymatic activity by titration in square-root 10 dilution steps on supercoiled plasmid DNA (two sites). Reactions were extracted with phenol/dichloromethane to remove DNA-bound protein, and analyzed by 1% agarose gel electrophoresis (upper panel, right and left). Neither subunit displayed detectable cleavage or nicking activity. ***Panel b****:* The two subunits were mixed 1:1, re-assayed in the same way (left), and the mixture displayed robust DNA-cleavage activity, indistinguishable from that of the stock BbvCI restriction enzyme (right). This new activity must stem from *de novo* R1:R2 enzyme molecules that arise by subunit association *in vitro*.

**
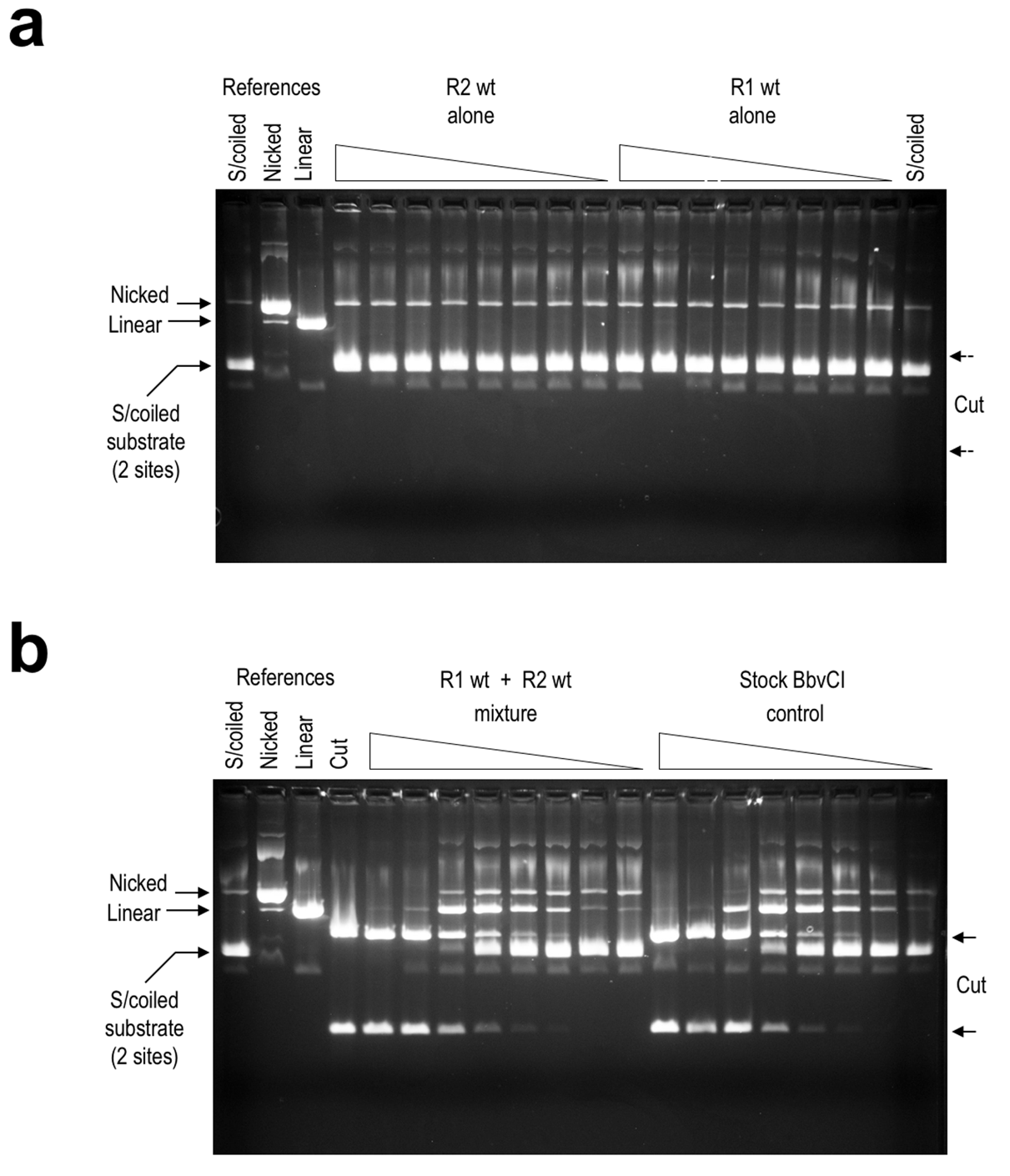
**

**Supplementary Figure S7. Panel a**: Recognition sequence similarities among Type II restriction enzymes Bsu36I, BlpI, Bpu10I, and BbvCI. Bsu36I and BlpI act as homo-multimers and recognize symmetric DNA sequences. Bpu10I and BbvCI act as hetero-multimers and recognize asymmetric sequences, the left half of which is Bsu36I-like, and the right half of which is Blp-like. We speculate that Bpu10I might have arisen as a chimaera between ancestral Bsu36I and BlpI subunits, and that BbvCI diverged from this by acquiring specificity for the central base pair. **Panel b**: Amino acid sequence alignment and co-variation among the subunits of these enzymes. Highlighted regions are mentioned in the text.

**
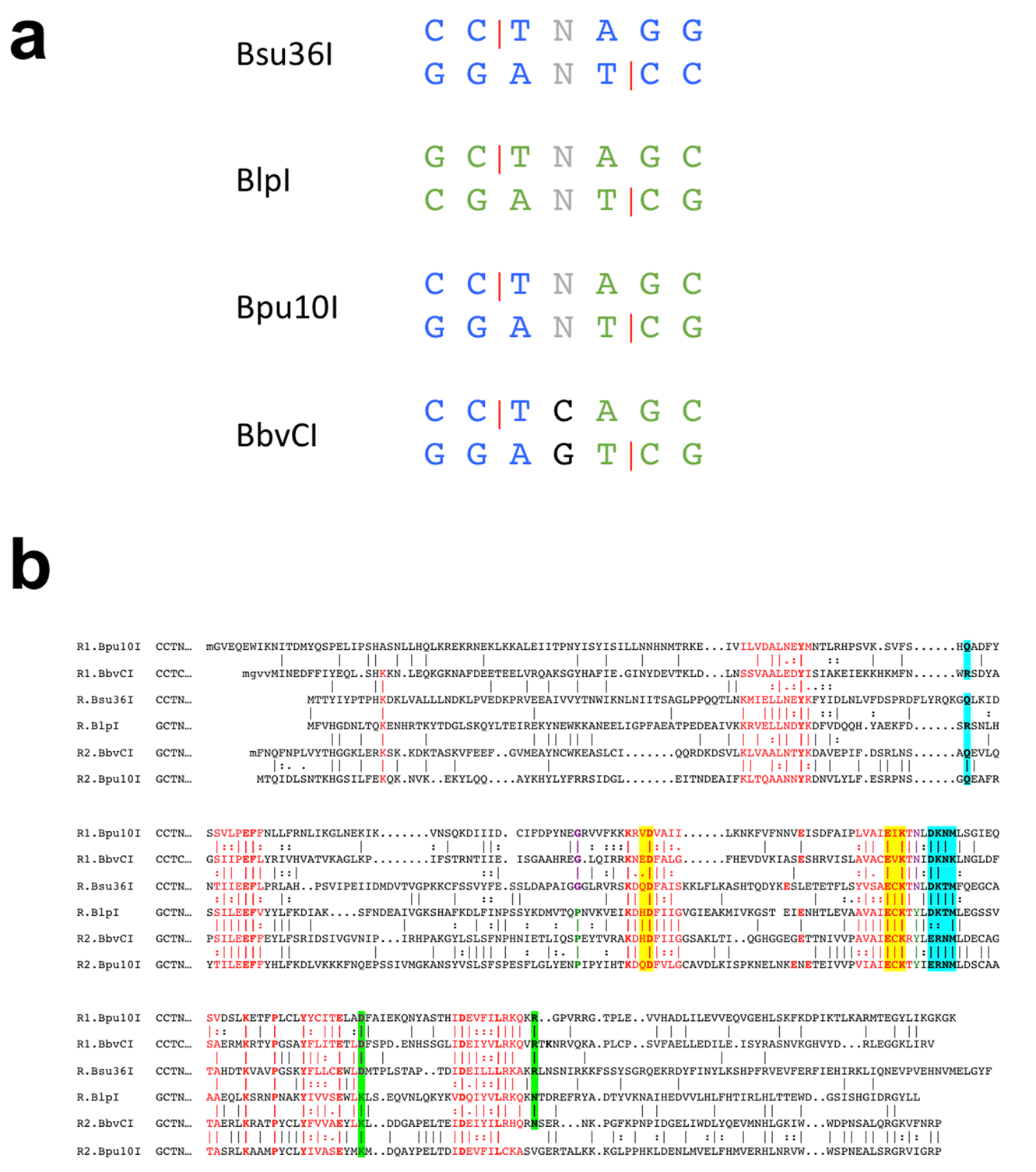
**

**Supplementary Table S1.** Structure-based analysis of the buried subunit interfaces throughout the complete tetramer. Out of the entire calculated buried interfacial surface area and corresponding free energy of association for the tetramer (5094 Å^2^; -37 kcal/mol), a substantial contribution is made by the R2-R2 interface (1838 Å^2^; -32 kcal/mol), whereas all other interfaces in the tetramer contribute about 600 to 900 Å^2^ that are predicted to add a few extra kcals/mol of association energy. In particular, the R1-R2 interface that spans the putative DNA-binding cleft and two active sites is quite minimal in buried surface area and actually is predicted to be unstable on its own (+4 kcal/mol calculated free energy of association). The relationshiop between interfaces in the table is indicated pictorially in the panels below.

**Interface Surface Area ΔG_calc_ # Potential H-bond # Potential Salt-Bridge**

Full tetramer 5094 Å^2^ -37 kcal/mol 28 4

R1-R1 847 Å^2^ -6 kcal/mol 10 2

R2-R2 1838 Å^2^ -32 kcal/mol 10 2

R1-R2 (A)* 574 Å^2^ +4 kcal/mol 2 0

R1-R2 (B)* 625 Å^2^ -8 kcal/mol 2 0

* Each of the two distinct interfaces formed between R1 and R2 subunits in the complete heterotetrameric assemblage is repeated twice (related by dyad symmetry), doubling all values within the complete holoenzyme structure.


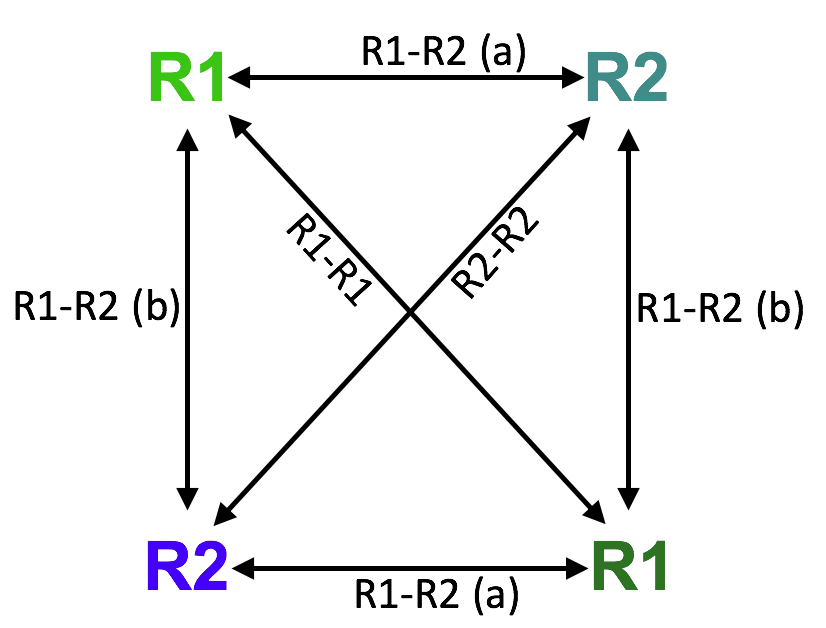

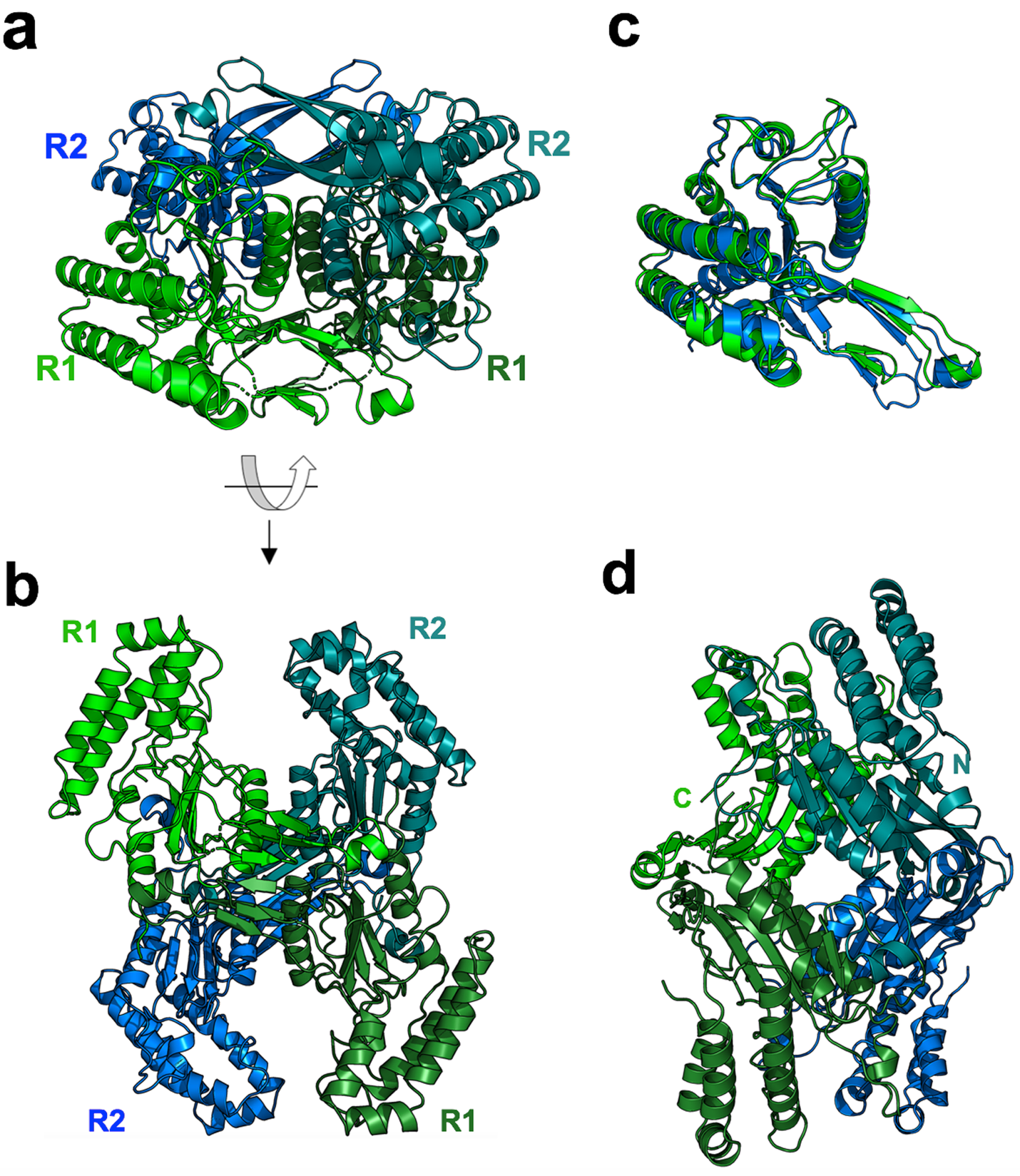

Supplement: Supplementary Data [file gky1059_supplemental_files.docx]
